# Metagenomics combined with activity-based proteomics point to gut bacterial enzymes that reactivate mycophenolate

**DOI:** 10.1080/19490976.2022.2107289

**Published:** 2022-08-11

**Authors:** Joshua B. Simpson, Josh J. Sekela, Amanda L. Graboski, Valentina B. Borlandelli, Marissa M. Bivins, Natalie K. Barker, Alicia A. Sorgen, Angie L. Mordant, Rebecca L. Johnson, Aadra P. Bhatt, Anthony A. Fodor, Laura E. Herring, Hermen Overkleeft, John R. Lee, Matthew. R. Redinbo

**Affiliations:** a Department of Chemistry, University of North Carolina at Chapel Hill, Chapel Hill, NC, USA; b Department of Pharmacology, University of North Carolina at Chapel Hill, Chapel Hill, NC, USA; c Department of Bioorganic Synthesis, Leiden Institute of Chemistry, Leiden University, Leiden, The Netherlands; d UNC Proteomics Core Facility, Department of Pharmacology, University of North Carolina at Chapel Hill, Chapel Hill, NC, USA; e Department of Biological Sciences, University of North Carolina at Charlotte, Charlotte, NC, USA; f Center for Integrative Chemical Biology and Drug Discovery, Division of Chemical Biology and Medicinal Chemistry, UNC Eshelman School of Pharmacy, University of North Carolina at Chapel Hill, Chapel Hill, NC, USA; g Division of Gastroenterology and Hepatology, Department of Medicine, Center for Gastrointestinal Biology and Disease, and the Lineberger Comprehensive Cancer Center, University of North Carolina at Chapel Hill, Chapel Hill, NC, USA; h Department of Bioinformatics and Genomics, University of North Carolina at Charlotte, Charlotte, NC, USA; i Department of Medicine, Division of Nephrology and Hypertension, New York, New York, USA; j Department of Biochemistry and Biophysics, Department of Microbiology and Immunology, and the Institute for Biological and Genome Sciences, University of North Carolina at Chapel Hill, Chapel Hill, NC, USA

**Keywords:** Multi-Omics, Metagenomics, Proteomics, Metaproteomics, Microbiome, Glycoside Hyrolase, Beta-Glucuronidase, Immunosuppression, Mycophenolate Mofetil

## Abstract

Mycophenolate mofetil (MMF) is an important immunosuppressant prodrug prescribed to prevent organ transplant rejection and to treat autoimmune diseases. MMF usage, however, is limited by severe gastrointestinal toxicity that is observed in approximately 45% of MMF recipients. The active form of the drug, mycophenolic acid (MPA), undergoes extensive enterohepatic recirculation by bacterial β-glucuronidase (GUS) enzymes, which reactivate MPA from mycophenolate glucuronide (MPAG) within the gastrointestinal tract. GUS enzymes demonstrate distinct substrate preferences based on their structural features, and gut microbial GUS enzymes that reactivate MPA have not been identified. Here, we compare the fecal microbiomes of transplant recipients receiving MMF to healthy individuals using shotgun metagenomic sequencing. We find that neither microbial composition nor the presence of specific structural classes of GUS genes are sufficient to explain the differences in MPA reactivation measured between fecal samples from the two cohorts. We next employed a GUS-specific activity-based chemical probe and targeted metaproteomics to identify and quantify the GUS proteins present in the human fecal samples. The identification of specific GUS enzymes was improved by using the metagenomics data collected from the fecal samples. We found that the presence of GUS enzymes that bind the flavin mononucleotide (FMN) is significantly correlated with efficient MPA reactivation. Furthermore, structural analysis identified motifs unique to these FMN-binding GUS enzymes that provide molecular support for their ability to process this drug glucuronide. These results indicate that FMN-binding GUS enzymes may be responsible for reactivation of MPA and could be a driving force behind MPA-induced GI toxicity.

## Introduction

The prodrug mycophenolate mofetil (MMF; CellCept) was approved by the FDA in 1995 and is now widely prescribed to prevent organ transplant rejection and to treat autoimmune diseases.^1,2^ Mycophenolic acid (MPA), the active form of MMF, inhibits the lymphocyte isoform of inosine monophosphate dehydrogenase (IMPDH), thereby arresting the proliferation of B and T lymphocytes by limiting their synthesis of guanosine triphosphate (GTP).^2,3^ Treatment with MMF, however, is often limited by severe GI side effects that are observed in approximately 45% of recipients.^4,5^

Recent studies have demonstrated that treatment with MMF initiates a shift in the microbiome, and that these changes may mediate drug-associated GI toxicity.^6–8^ Indeed, GI toxicity was dramatically reduced in germ-free mice compared to controls when treated with MMF.^6^ Additionally, treatment with the antibiotic vancomycin was shown to ameliorate GI toxicity in mice administered MMF, establishing the microbiota’s involvement in this unwanted side effect.^8^ When a human subject was administered antibiotics alongside MMF; however, GI toxicity was not alleviated.^7^ Given that organ transplant recipients are typically administered MMF over extended periods, an antibiotic-directed approach is likely not a practical therapeutic approach for severe GI toxicity, as the microbiota also play critical roles in metabolism and homeostasis.^5,9–13^

**Figure 1.**
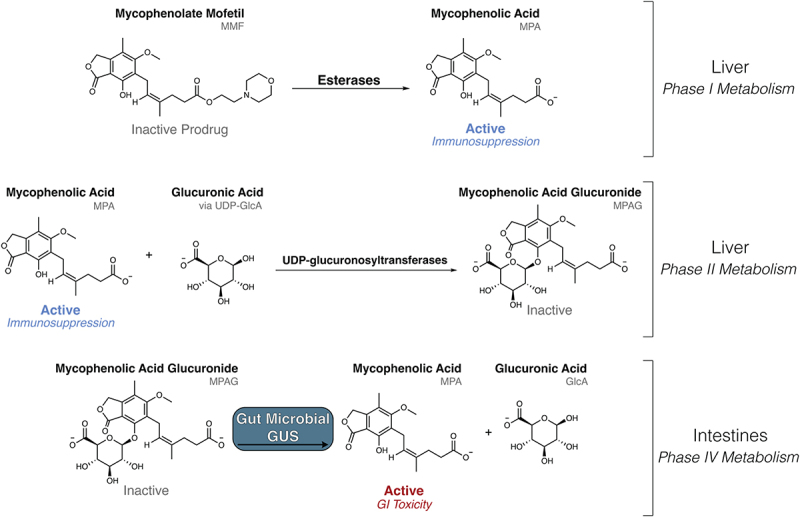
The orally administered MMF is activated by host esterases to mycophenolic acid (MPA), an immunosuppressant that impedes DNA synthesis in B and T lymphocytes. Liver MPA is inactivated via glucuronidation to mycophenolic acid glucuronide (MPAG) by UDP-glucuronosyltransferases (UGTs) and sent to the intestines for excretion. Gut microbial β-glucuronidase (GUS) enzymes remove the glucuronide as a source of carbon, and active MPA is reabsorbed in the gut lumen, contributing to gastrointestinal toxicity.

MMF is ester-linked with a morpholino ethyl moiety that increases efficacy and bioavailability; this group is removed by host esterases to produce active MPA, which interacts with the microbiota through so-called phase IV metabolism (Figure 1).^1,14,15^ MPA is glucuronidated in the liver by uridine diphosphate (UDP)-glucuronosyltraerase enzymes to form inactive mycophenolate glucuronide (MPAG), which is excreted via the biliary ducts to the gastrointestinal tract.^15^ Once in the intestines, MPA can be reactivated by bacterial β-glucuronidase (GUS) enzymes (Figure 1). Indeed, mice that received both MMF and vancomycin showed decreased fecal levels of active MPA and higher fecal levels of inactive MPAG compared to mice only receiving MMF, suggesting that gut microbes extensively metabolize MPAG.^8^ MPA that is reactivated in the gut can be reabsorbed into systemic recirculation, altering therapeutic levels of this critical immunosuppressant and exacerbate neutropenia, which is an indicator of excessive neurosuppression.^8,16^ Within the gut, MPA reduces the integrity of the GI epithelial barrier, resulting in side effects such as vomiting, diarrhea, ulcers, and decreased concentrations of short-chain fatty acids.^5,17,18^ Thus, gut microbial GUS enzymes play a crucial role in the many deleterious side effects of MMF.

GUS enzymes reactivate a variety of xenobiotic and endobiotic glucuronides from their inactive glucuronide metabolites, including cancer drugs irinotecan and regorafenib, several NSAIDs, the consumer antimicrobial compound triclosan, as well as several forms of estrogen.^19–23^ Prior metagenomic analyses have identified hundreds of putative GUS enzymes that can be categorized into one of eight unique structural classes displaying distinct substrate preferences.^24–26^ The development of a GUS-specific activity-based chemical probe has enabled the use of targeted metaproteomics to quantify the GUS proteins present and active in human fecal samples.^23,27^ This technology has been further adapted to identify the structural classes of GUS enzymes that are responsible for reactivation of irinotecan and triclosan.^21,23^

While gut microbial GUS enzymes likely reactivate MPA, the structural details of this reactivation reaction have not been explored toward identifying which of the hundreds of potential GUS orthologs may be primarily responsible. We hypothesized that bacterial GUS abundance and composition would vary between transplant recipients receiving MMF and healthy individuals, and that these differences would enable us to pinpoint the specific GUS enzymes responsible for MPA reactivation. Here, we compare the microbiomes and metagenomic GUS profiles of transplant recipients receiving MMF to healthy individuals using shotgun metagenomic sequencing. We then analyze the fecal samples using GUS-targeted metaproteomics leveraging the cohort’s metagenomics data as a reference. The results obtained show that, while metagenomic sequencing data are insufficient to show correlations with MPA reactivation rates, the metaproteomics data revealed clear associations with the levels of FMN-binding GUS proteins and the rates of drug reactivation in individual samples. Finally, we explore the potential structural basis of these observations with a panel of purified GUS enzymes. Together, these data suggest for the first time that specific FMN-binding GUS enzymes may be responsible for MPA-induced GI toxicity.

## Results

### MMF treatment and gut microbial composition

We collected fecal samples from five renal transplant recipients receiving MMF and four healthy individuals. The transplant recipients had ages ranging from 43 to 79 y, and all were receiving a calcineurin inhibitor-based maintenance regimen as well as MMF. Further details of the transplant recipients are found in Supplemental Table 1. In four cases, the transplant recipient fecal samples were collected closely following transplantation (d 4–9), but in one case (T3) it was collected 227 d after the transplant. The healthy individuals had ages ranging from 22 to 52 y and had not received antibiotics for several months prior to fecal collection. All individuals, both transplant recipients and controls, were male.

**Figure 2.**
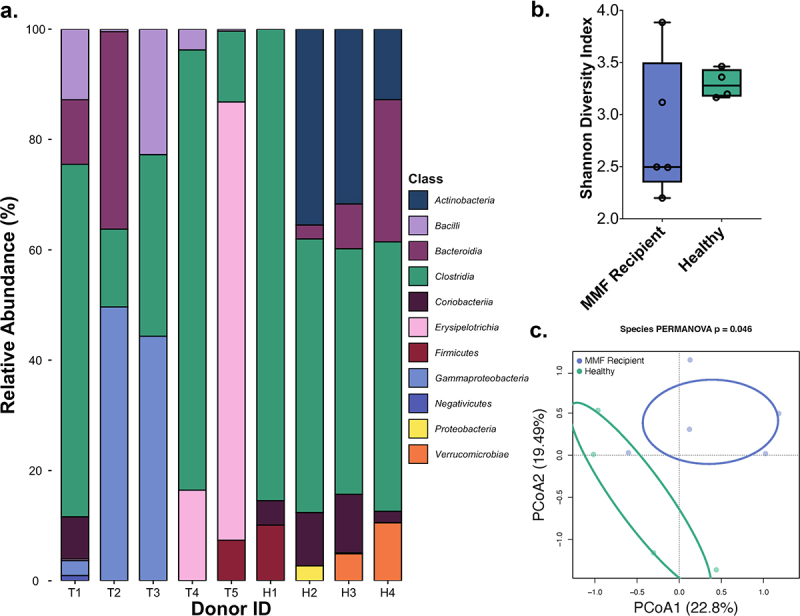
Metagenomic Shotgun Sequencing profiles for MMF recipients (blue; T1-T5) and healthy individuals (green; H1-H4). (a) Relative abundance of intestinal bacteria by Class. (b) Alpha diversity by the Shannon Diversity Index. (c) Bray Curtis PCoA ordination of Beta diversity at the species level from shotgun metagenomic sequencing and shown with PC1 and PC2 components.

We first examined the fecal metagenomic profiles using shotgun metagenomic sequencing (Supplemental Figure 1). Results at the class level of taxonomies are shown in Figure 2a, while phyla, order, family, genus, and species levels of taxonomies are shown in Supplemental Figures 3–7, respectively. While all samples contained microbes of the *Clostridia* class, distinct class differences were observed between groups. Specifically, the flora of transplant recipients who received MMF had the presence of *Bacilli, Gammaproteobacteria*, and *Erysipelotrichia*, which were not observed in healthy individual samples; in contrast, healthy individuals uniquely had the presence of *Actinobacteria* and *Verrucomicrobiae* (Figure 2a).

There was similar alpha diversity between the fecal samples from MMF-treated transplant recipients and those from healthy individuals as measured by the Shannon Diversity Index (Figure 2b). However, the beta diversity at the species level between transplant recipients and healthy individuals was different (Figure 2c; *P* = .046), and these differences did not appear to be a function of time elapsed since kidney transplantation (Supplemental Figure 2; Supplemental Table 1).

### Stool metagenomic gusome profiles do not differ between kidney transplant recipients who received MMF and healthy individuals

**Figure 3.**
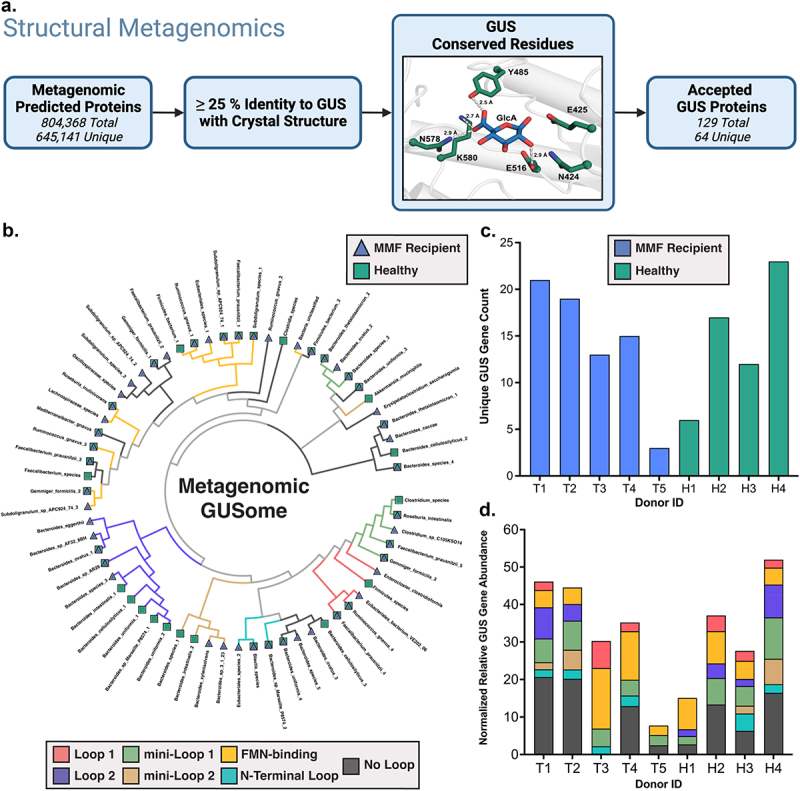
(a) Structural metagenomics workflow for identification of GUS proteins. (b–d) Metagenomic GUS gene profiles for MMF recipients (blue; T1–T5) and healthy individuals (green; H1–H4). (b) Cladogram reflecting non-redundant GUS gene sequences across the cohort. Each node represents GUS protein sequences with >90% identity. GUS class and treatment groups from which genes were derived are indicated. Unique GUS gene counts for each donor (c) and normalized gene abundances clustered by GUS class (d) are also shown. Figure 3a was created with BioRender.com.

The gene catalogs extrapolated from shotgun metagenomics for our cohort contained 884,299 total translated protein sequences, and we employed a structure-guided approach to identify those that encoded genes for GUS enzymes (Figure 3a; Supplemental Figure 1). Each sequence was aligned to the 17 representative gut microbial GUS enzymes with reported crystal structures; sequences with >25% identity to at least one representative GUS enzyme were then assessed for the presence of 7 conserved active site residues essential for glucuronide hydrolysis.^24^ Predicted protein sequences that met both the identity threshold and contained all conserved residues were accepted as GUS enzymes. Taxonomy of origin and GUS structural class for each sequence were then assigned by comparing protein sequences to the RefSeq Select database. In total, 130 genes for GUS proteins were detected, and clustering into groups with >90% sequence identity collapsed these into a final collection of 64 GUS proteins (a “GUSome” for this dataset; Figure 3b). Nearly all the sequences are derived from either *Bacteroidetes* or *Firmicutes* phyla (Supplemental Figure 8).

We then turned to the distinct structural clades that have been assigned to gut microbial GUS enzymes.^24–26^ Several clusters within the metagenomic GUSome belonging to a structural class were found to be either uniquely present or notably missing from the fecal samples taken from transplant recipients receiving MMF. Notably, *Oscillospiraceae*-derived “No Loop” GUS genes are only found within MMF recipients, while genes for *Bacteroidaceae*-derived “Loop 2” GUS enzymes are entirely absent from this group (Figure 3b). There were other instances of individual GUS genes within a structural class being present within only one treatment group. However, we observed no differences between MMF recipients and healthy individuals in overall GUS gene abundance overall or for any structural class (Supplemental Figure 9).

The number of unique GUS genes ranged from 3 to 21 in the samples from MMF-treated transplant recipients, and 6 to 23 in samples from healthy individuals (Figure 3c). These ranges are akin to those observed earlier in the 139 donor samples from the Human Microbiome Project stool sample database, which showed a range of 4–41 unique GUS proteins per individual (and termed an individual’s “GUSome”).^24^ Similarly, the individual GUSomes of each sample in the current study exhibited a range of gene abundances for GUS enzymes of different structural classes (Figure 3d). While the samples from MMF-treated transplant recipient T5 contained genes from only three GUS classes, several samples contained genes for all seven classes (T1, H3, H4) or six of seven (T2). Together, these results suggest that the metagenomic profiles of the MMF-treated transplant recipients and healthy individuals contain genes coding a similar range of structurally diverse GUS proteins.

### Differences in mycophenolate reactivation between fecal samples

**Figure 4.**
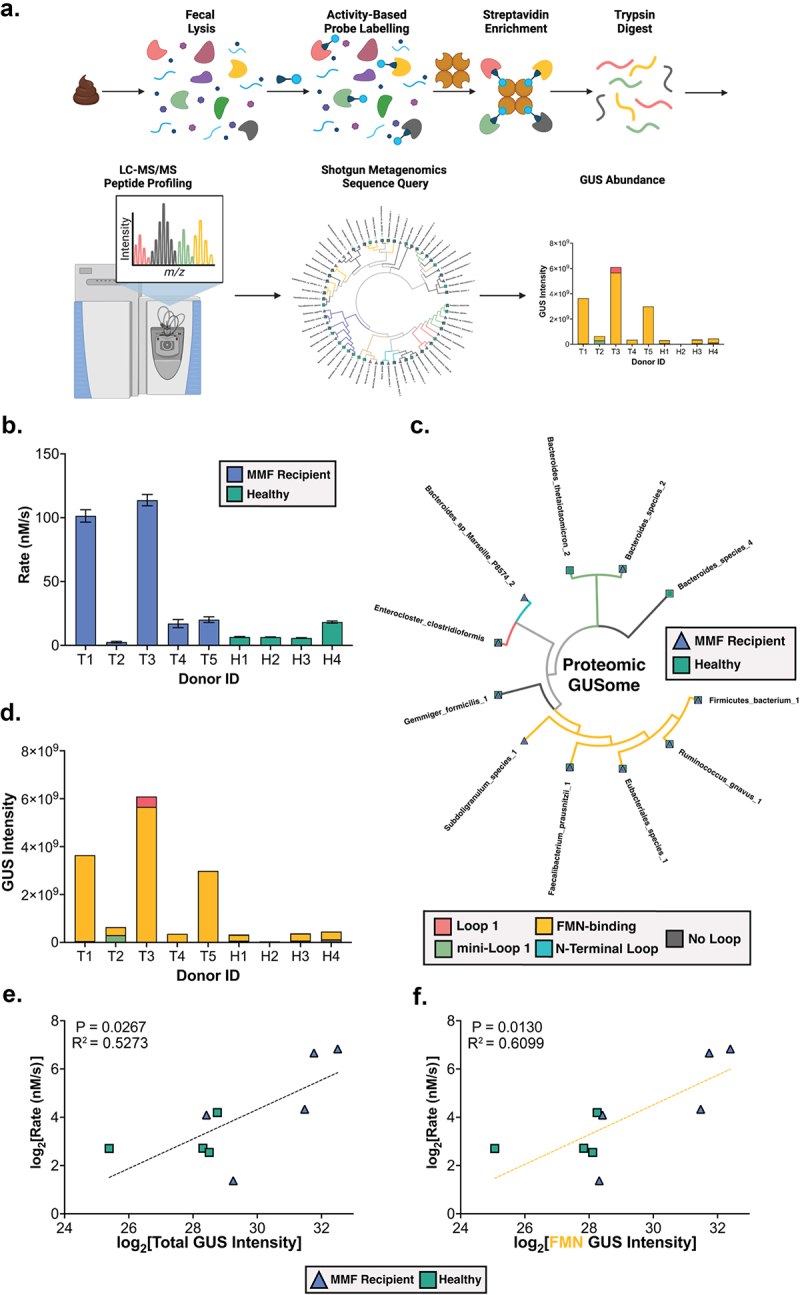
(a) Activity-based probe-enabled proteomic pipeline. (b) *Ex vivo* reactivation of MPAG by donor fecal extracts; data reflect the mean of three biological replicates and error bars reflect SEM. (c) Cladogram reflecting GUS proteins identified across cohort. GUS class and treatment groups from which proteins were derived are indicated. (d) Proteomic profiles for GUS proteins in MMF recipients and healthy individuals. Metaproteomic GUS abundance is represented by Intensity, which is the combined peptide signal intensity corresponding to each reference GUS sequence from Shotgun Metagenomic Sequencing. Proteins were binned according to GUS class. (e–f) Correlation analysis between MPAG reactivation rate and normalized total GUS protein abundance (E; *P* = .027) or FMN GUS protein abundance (F; *P* = .013). *P* values reflect confidence in a slope that is significantly non-zero as determined by the Wald test. Figure 4a was created with BioRender.com.

Given that shotgun metagenomics reflects only the genes present in the microbiome and not necessarily the proteins that are expressed and active, we sought to explore the potential differences in bulk GUS enzyme activity toward mycophenolate-glucuronide hydrolysis between fecal samples collected from the five renal transplant recipients receiving MMF and the four healthy individuals. To do so, we extracted the complex protein lysates from each fecal sample and measured the rate of reactivation of MPA from the inactive metabolite MPAG. This approach has been employed previously to examine drug and toxin reactivation rates by fecal lysates and to correlate these values with meta-proteomic GUS abundances.^21,23^ Here, we found MPA reactivation rates between 3 and 114 nM/s for MMF-treated transplant recipients’ samples, and between 6 and 18 nM/s for the healthy individual samples (Figure 4b). Thus, a range of values were observed across all samples, and the highest rates recorded were from MMF-treated transplant recipient samples. These observations could not be generalized to significant differences between treatments given the wide range in reactivation rates across individuals in both groups. However, the >20-fold differences in MPA reactivation rates between samples did not correlate with abundance of microbial class, phylum, family, genus, or species (Supplemental Table 4), or with any feature of the metagenomic GUSome profiles outlined in Figure 3 (Supplemental Figure 10), with one exception at the species level. The relative abundance of *Streptococcus parasanguinis*, a species only present in transplant recipients, correlated with rate of MPA reactivation with a slope that was significantly non-zero as determined by the Wald test (*P* = .0486; Supplemental Table 4). However, the relative abundance of this species ranged from just 1% to 7%, and GUS genes from this species were not detected. Thus, metagenomic sequencing data, even when examined at the level of GUS protein functional classes, are insufficient to explain the differences in MPA reactivation rates observed within or between transplant recipient and control samples. This observation suggests that differences in the expression or abundance of specific gut microbial GUS proteins between samples may provide this explanation.

### MMF treatment and gut bacterial GUS protein composition

Next, we sought to explore the metaproteome of GUS enzymes between the fecal samples collected from the five kidney transplant recipients receiving MMF and from the healthy individuals. The protein lysates from each fecal sample were examined by activity-based probe-enabled proteomics using a biotin-linked covalent probe for GUS enzymes, which we have adapted from our previous studies using human fecal samples and therapeutic reactivation rates, and the reactivation of the antimicrobial compound triclosan.^21,23^ The proteomics pipeline outlined in Figure 4a was followed to output peptide fragments that were then used to identify individual GUS proteins.^21,23^ Importantly, though, the current study uses the protein sequences derived from shotgun metagenomics collected from these exact fecal samples (Figures 2 and 3) as the reference database to identify GUS proteins, rather than a standard reference metagenome like the Integrated Gene Catalog (IGC).^28^ In doing so, we found that GUS proteome percent peptide coverage was significantly improved as determined by a Wilcoxon matched-pairs signed rank test (*P* = .031; Supplemental Figure 11) using the cohort-specific metagenomics data compared to the IGC as a reference database.

GUS proteins of all structural classes that were detected in metagenomics were also present in the probe-enabled proteomics data, except for Loop 2 and mini-Loop 2 (Figure 4c). Eleven unique GUS proteins were detected in total, with most GUS proteins being present in both groups (Figure 4c). All samples contained GUS proteins, with FMN-binding GUS enzymes being detected the most frequently across the cohort (Figure 4d). Notably, samples from transplant recipients receiving MMF contained significantly more GUS proteins than healthy individuals when compared by Welch’s *t*-test (*P* = .034; Supplemental Figure 12). We then compared abundance of GUS proteins by structural class and found that FMN-binding GUS enzymes are significantly elevated in transplant recipients receiving MMF compared to healthy individuals as determined by a Welch’s *t* test (*P* = .029; Supplemental Figure 12). In addition, “No Loop” GUS enzymes were abundant in the four healthy individual samples but were not detected in four of the five MMF recipients (*P* = .009; Supplemental Figure 12). Outside of FMN-binding and “No Loop” GUS enzymes, there were no other significant differences in GUS abundances by structural class between treatment groups (Supplemental Figure 12). Thus, activity-based probe-enabled proteomics demonstrate that the fecal samples from transplant recipients who received MMF showed increased levels of FMN-binding GUS enzymes compared to fecal samples from healthy individuals.

To directly evaluate the impact that these differences in GUS abundance might have on MPAG processing in the gut, we sought to identify trends between MPA reactivation rates and GUS composition and/or abundance by sample. First, we found that reactivation linearly increases with overall abundance of GUS proteins regardless of treatment group with a correlation that is significantly non-zero by the Wald test (*P* = .027; Figure 4e). We then mapped abundance of GUS proteins by structural class to rate of MPA reactivation. Strikingly, the abundance of FMN-binding GUS enzymes also strongly correlates with rate of MPAG reactivation with a slope that is significantly non-zero by the Wald test (*P* = .013; figure 4f). No significant correlations were observed between proteomic levels of other GUS structural classes and MPA reactivation (Supplemental Figure 13). Together, these results indicate that FMN-binding GUS enzymes are associated with reactivation of MPA in human fecal samples.

### FMN-Binding GUS active site environment and MPAG

**Figure 5.**
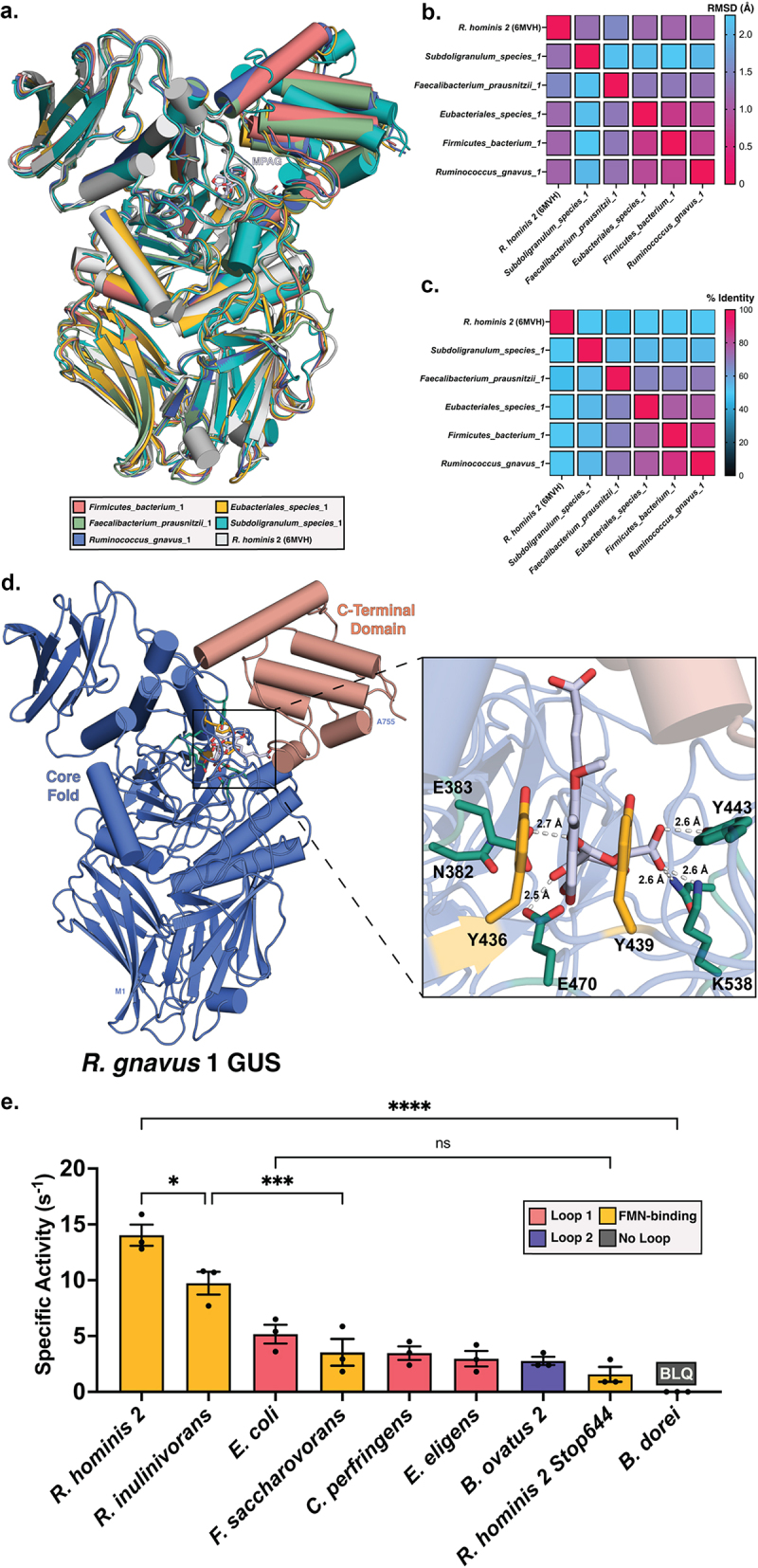
Comparison of AlphaFold models for five meta-proteome derived FMN-binding GUS enzymes with an x-ray crystal structure model for the FMN-binding GUS enzyme *R. hominis* 2. (a) Overlay of seven structures. (b) Heatmap comparing Root-mean-square deviation (RMSD) in Angstroms for the six structures. (c) Heatmap comparing percent sequence identity for the sequences of the six structures. (d) Active site analysis of *R. gnavus* 1 GUS monomer (blue) with C-terminal domain (CTD) shown in coral. Catalytic residues are shown in green, aromatic residues conserved across FMN-binding GUS enzymes are shown in yellow, and MPAG is shown in gray. (e) Specific activities for a panel of purified GUS enzymes. Colors of bars correspond to structural class. Averages represent the averages of three biological replicates (shown as individual points) ± SEM. Rates were compared using Tukey’s multiple comparisons test (*P < .05, ***P < .001, ****P < .0001, ns = non-significant).

To identify a structural rationale for the strong positive correlation of abundance of FMN-binding GUS enzymes with MPA reactivation rate, we used AlphaFold to model protein structures for the five FMN-binding GUS enzymes identified by the activity-based probe-enabled proteomics pipeline employed here.^29^ We overlaid these five structures with the extant structure of the FMN-binding GUS enzyme from *Roseburia hominis* 2 (*Rh*2GUS) which was resolved with x-ray crystallography (Figure 5a).^26^ All core protein domains were heavily conserved between the crystal structures of FMN GUS enzymes and our AlphaFold models, which is reflected in the RMSD values ranging from 0.5 Å to 2.3 Å (Figure 5b). The C-terminal domain (CTD) flanking the enzyme active site is the area with the greatest variance in positioning between our GUS enzyme models, which is expected as this domain has yet to be resolved in a crystal structure of an FMN-binding GUS. Overall, the models are similar, as reflected in the relatively low RMSDs and high percent identities for these FMN-binding GUS enzymes identified in the metaproteome (Figure 5b,c).

We next analyzed the active sites of our modeled FMN-binding GUS enzymes to identify residues that may be involved in the efficient hydrolysis of MPAG. MPAG was docked into the active sites of each of the modeled structures for the proteome-derived FMN-binding GUS enzymes. Intermolecular interactions predicted between MPAG and *R. gnavus* 1 GUS are shown in Figure 5d, which are representative of the interactions predicted between MPAG and the FMN-binding GUS enzymes within our metaproteomics data. Two consistent interactions were then noted across the substrate-enzyme complexes. First, the MPA core scaffold is positioned between two aromatic residues favorable for π-π stacking interactions. Importantly, while tyrosine 439 is conserved across all GUS enzymes, the aromatic moiety of tyrosine 436 is only conserved across the FMN-binding GUS enzymes detected in our metaproteomics data and not across GUS enzymes from other structural classes within these data. Second, the carboxylic acid tail of MPA is positioned approximately 3 Å from the CTD (shown in coral in Figure 5d), providing conditions favorable for salt bridges or van der Waals interactions between the carboxylic acid and residues of the CTD. Together, these structural models indicate that aromatic residues conserved in the active sites of FMN-binding GUS enzymes detected in our metaproteomics data provide π-π stacking interactions that may efficiently facilitate MPA reactivation by these gut microbial proteins.

### Differences in MPA reactivation between purified GUS enzymes

To provide support for our *in silico* modeling hypotheses, we assessed the ability of a panel of purified GUS enzymes to reactivate MPA. Specifically, we determined specific activities for purified GUS enzymes derived from reference bacterial genomes across the IGC that exhibit GUS activity with other glucuronidated substrates.^20–22,28^ The rate of MPA reactivation was significantly greater for two purified FMN-binding GUS enzymes from *Roseburia* hominis (*Rh*2GUS) and *Roseburia* inulinivorans (*Ri*GUS) than all other purified GUS enzymes by a Tukey’s multiple comparison test (Figure 5e; *p* < .05), with both FMN-binding GUS enzymes exhibiting at least twofold greater activity compared to all other structural classes across our panel. Notably, the FMN-binding GUS derived from *Fusobacterium saccharovorans* (*Fs*GUS) processed MPAG at a rate approximately threefold lower than *Rh*2GUS (*p* < .0001). However, one of two active site aromatics hypothesized to be involved with positioning MPAG (Figure 5d; Y436 in *R. gnavus* 1 GUS) is replaced by an isoleucine in *Fs*GUS. Similarly, Y436 is replaced by a leucine in all Loop 1 enzymes in our purified panel, suggesting that an aromatic residue in this position is indeed necessary for efficient reactivation of MPA. To further explore the structural features of FMN-binding GUS enzymes suggested by our modeling to contribute to efficient MPA reactivation, we removed the CTD from our most efficient purified enzyme, the FMN-binding *Rh*2GUS, by placing a stop codon after residue 644 (denoted *R. hominis* 2 Stop644 in Figure 5e).^20^ Strikingly, removing the CTD in *Rh*2GUS resulted in an eightfold reduction of specific activity compared to the wild-type enzyme (Figure 5e). While both *Fs*GUS and *R. hominis* 2 Stop644 could still process MPA to a limited extent, their specific activities were comparable to that of both Loop 1 and Loop 2 enzymes. Together, these results suggest that both the CTD and the aromatic residues surrounding the active site contribute to the efficient reactivation of MPA by FMN-binding GUS enzymes.

## Discussion

To identify the GUS genes within our shotgun metagenomics data, we applied a structure-guided approach toward our cohort’s predicted protein sequences (Figure 3a).^24,26^ We identified 64 unique GUS genes in total from 7 distinct structural classes, with the majority being derived from *Bacteroidetes* and *Firmicutes*. Several clusters of GUS genes of the same structural class were notably unique or absent in transplant recipients altogether (Figure 3b). We sought, then, to explain the range in MPA reactivation by fecal lysates using either overall microbiome composition or by metagenomic GUS profiles between samples or groups, as metagenomic gene abundances have historically been used to predict gene expression and functional potential.^8,30–33^ However, we were unable to find any correlations between the rates of drug reactivation measured and any compositional or GUS metagenomic feature (Supplemental Figure 10). We therefore concluded that, for this cohort, the fecal shotgun metagenomic data were insufficient to pinpoint specific gut microbial GUS enzymes that may have been responsible for the 38-fold difference in MPAG to MPA processing rates observed between fecal samples.

We then used activity-based probe-enabled proteomics to directly identify and quantify the GUS proteins present in our cohort samples (Figure 4a).^34^ Peptide fragments were matched to 11 unique GUS proteins, with most structural classes of GUS proteins being detected in both treatment groups, and FMN-binding GUS enzymes being the most prevalent (Figure 4c). Samples from transplant recipients receiving MMF contained more GUS proteins overall than healthy individuals (*P* =.034; Supplemental Figure 12). Furthermore, FMN-binding GUS enzymes were more abundant in MMF transplant recipient samples (*P* =.029), and “No Loop” GUS enzymes were not detected in the majority of MMF recipients (*P* =.0097).

When these findings are considered in the context of our metagenomics data, two aspects are noteworthy. First, while the metagenomics showed that samples from all members of the cohort except T3 contained at least one “No Loop” GUS gene, proteins from this structural class were only detected in one MMF recipient by metaproteomics. Conversely, “No Loop” GUS proteins were detected in both the metagenomics and the metaproteomics of all healthy individuals (Figure 3d; Figure 4d). In all previous studies quantifying GUS abundance with targeted metaproteomics, “No Loop” GUS enzymes were detected in all samples.^21,34^ Therefore, the intestinal environment of transplant recipients receiving MMF appears to shift in favor of FMN-binding GUS enzymes over “No Loop” GUS ortholog. Second, the 11 unique GUS proteins were identified using metaproteomics represent a small subset of the 64 unique GUS genes that could be expressed across the cohort. Our previous work has shown that all structural classes of GUS enzymes are detectable by our probe, so the differences are not driven by differential probe reactivity. Instead, our results suggest that only this subset of GUS enzymes were expressed with sufficient abundance to be detected in our probe-enabled proteomics pipeline (Figure 4a).^23^ By using the protein sequences derived from shotgun metagenomics collected from these fecal samples as the reference database to identify GUS enzymes (Figures 2 and 3), we significantly improved peptide coverage for matched proteins. Thus, while fewer unique proteins are abundant than are encoded within the metagenomic GUSome for our cohort (Figure 3b), the metaproteomics peptide coverage suggests high confidence matches for these 11 proteins.

When rates of MPA reactivation were compared to these abundances, we found a positive correlation between rate and overall abundance of GUS (Figure 4e). By further delineating this correlation according to individual structural classes of GUS, we identified FMN-binding GUS enzymes as the driving force behind this positive correlation. Indeed, when rates of MPA reactivation are compared to FMN-binding GUS abundance, we see the strongest positive correlation between abundance and rate of MPA reactivation for any class of GUS (figure 4f; Supplemental Figure 13). We attempted to relate the relative gene abundance for any structural class of GUS as determined by metagenomics with the protein abundance of the class as determined by metaproteomics, but no correlations were identified (Supplemental Figure 14). We extended our approach toward relating bacterial abundance at any taxonomic level with rates of MPA reactivation; again, though, no significant trends were present for bacteria containing GUS genes (Supplemental Table 4). Together, these results suggest that changes in FMN-binding GUS protein expression, but not the overall abundance of these GUS-expressing microbes, are responsible for reactivation of MPA. Indeed, treatment with MMF has been shown to broadly alter the gene expression profiles of bacteria within the gut.^35^ While metagenomic approaches provide an accurate map of the possible proteins within one’s microbiota and are uniquely valuable in their ability to improve specificity of metaproteomic peptide fragment mapping, neither the relative abundance of bacteria or the abundance of genes should be used as a metric to predict GUS protein expression or activity.

Our study is the first to apply GUS-targeted metaproteomics toward analyzing a cohort who routinely use MMF. By doing so, we highlighted stark differences between the GUS profiles of these groups, suggesting that GUS abundance is increased in renal transplant recipients, and that FMN-binding GUS enzymes are the driving force behind the reactivation of MPA. All members of the transplant recipient cohort contain at least one FMN-binding GUS in their metaproteomics data, and the rate of MPA reactivation increases with abundance of FMN-binding GUS enzymes. However, samples from healthy individuals have a lower abundance of FMN-binding GUS enzymes and generally reactivate MPA less efficiently compared to the samples from MMF recipients. In all prior studies that apply activity-based proteomics toward discovering structural classes of GUS responsible for drug reactivation, “Loop 1” GUS enzymes were most strongly correlated with increased rate of reactivation.^21,34^ Some classes of GUS enzymes were sparsely represented across our cohort as a whole (Figure 4; Supplemental Figure 11), with no “Loop 2” GUS enzymes being detected and with “Loop 1” GUS enzymes only being detected in four of nine samples. The increased abundance of FMN-binding GUS enzymes in MMF recipients relative to healthy individuals supports our conclusion that FMN-binding GUS promote MPA reactivation in the gut, which may in turn contribute to GI toxicity and systemic recirculation of active MPA. Importantly, it is unclear whether this increased abundance of microbial FMN-binding GUS enzymes is directly a result of either kidney transplantation or the administration of MMF, or whether the duration of MMF treatment influences the abundance of these enzymes. Future studies will be directed toward obtaining baseline fecal samples from kidney transplant recipients prior to receiving MMF and then again after receiving MMF to further explore the changes in gut microbial GUS enzymes.

Finally, to pursue a structural rationale for the strong positive correlation of abundance of FMN-binding GUS with MPA reactivation, we modeled the proteomic GUSome FMN-binding GUS enzymes using AlphaFold, overlaid the structures with extant structures of FMN-binding GUS enzymes that were resolved with x-ray crystallography (Figure 5a), then explored the model-informed observations by assessing MPA reactivation for a panel of representative GUS enzymes (Figure 5e).^29^ Each of our models position the flexible CTD directly outside of the active site, where it may act as a “gate” for glucuronidated substrates. Given that removal of the CTD diminishes all GUS activity with MPA for *Rh*2GUS (Figure 5e), these initial interactions between the CTD and substrate are likely integral to MPAG recruitment and positioning for hydrolysis.^20,21,23^ Within the active site, and where the accuracy of the AlphaFold models is expected to be the highest, MPAG likely interacts via π-π stacking with two aromatic residues residing above the back of the glucuronic acid catalytic site (yellow; Figure 5d). While one of these aromatic residues (Y439 in *R. gnavus* 1 GUS) is conserved across all GUS enzymes, this second aromatic residue (Y436 in *R. gnavus* 1 GUS) is notably not conserved in the “No Loop” GUS enzymes that are closest to FMN-binding GUS proteins by sequence identity. The positioning of these aromatic residues is similarly reflected in the sequences of all other FMN-binding GUS enzymes across our cohort’s proteome, corroborating the accuracy of our AlphaFold models in this region. Indeed, the purified FMN-binding GUS enzymes containing both active site aromatics and their CTD more efficiently reactivate MPA compared to GUS enzymes of other structural classes (Figure 5e; *P* < .05). In the wild-type sequence for the FMN-binding *Fs*GUS, Y436 is substituted with a nonpolar isoleucine, and the protein exhibits a threefold reduction of MPA reactivation *in vitro* compared to *Rh*2GUS. Similarly, all Loop 1 enzymes in our panel of purified enzymes contain a leucine at this position and their rates of specific activity are comparable to *Fs*GUS. These observations suggest that MPAG is efficiently reactivated by FMN-binding GUS enzymes due to conditions favorable for π-π stacking at the active site that are facilitated in part by the CTD of these proteins. Future studies will further explore the molecular rationale of MPA reactivation by FMN-binding GUS enzymes.

MMF is an important immunosuppressive agent administered to organ transplant recipients and is widely prescribed to treat autoimmune disorders, with many transplant recipients requiring long-term drug administration. Here, we show that fecal samples with a greater abundance of FMN-binding GUS enzymes, particularly those from MMF-treated transplant recipients, reflected a faster rate of reactivation of MPA compared to healthy individuals. Together, our findings demonstrate that gut microbial FMN-binding GUS enzymes efficiently reactivate MPA, which may play a significant role in MPA-induced GI toxicity. The data presented in this study reinforce the relevance of the microbiome in MPA-induced toxicity. However, there are several limitations to this study. First, the sample size is small, which was by design to validate our proteomics pipeline with an initial set of patient and non-patient samples. A larger cohort of samples will be required to further validate our findings. Second, the patient samples were collected in New York, while the non-patient samples were collected in North Carolina, and difference in geography can affect human gut microbiomes.^36–39^ Third, the patients were suffering from kidney failure prior to transplant, and then had in most cases just undergone the transplant surgery. Both factors are likely to influence the structure and activity of the gut microbiome, as will their associated use of therapeutics beyond mycophenolate. Fourth, the fecal samples were not handled after collection to allow for transcriptomics analysis toward determining whether transcript levels might correlate with fecal MPA reactivation rates. Finally, all subjects were male to match MMF-recipient with MMF-recipient characteristics but leaving sex as a biological variable unexamined. Despite these limitations, several clear conclusions can be drawn from the data collected, most notably that the proteomics provided correlations with drug reactivation that the metagenomics did not. Thus, with additional validation and development, the data presented here may lay the groundwork for the identification of transplant recipients at-risk for MPA-induced gut damage or, by extending the established concept of targeted microbial GUS inhibitors, toward alleviating the GI toxicity in patients receiving MMF therapy.

## Method details

### Fecal sample collection

From May 2019 to January 2020, five male kidney transplant recipients were enrolled for collection of fecal specimens. The Weill Cornell Institutional Review Board approved the study (IRB#1207012730) and all transplant recipients provided written informed consent. Fecal samples were also collected from four healthy male volunteers at the University of North Carolina at Chapel Hill (IRB#17-1528). Fecal samples were collected using a toilet specimen collection kit (Fisher Scientific) and were stored at −80°C until further use. Demographics of the cohort can be found in Supplemental Table 1.

### Preliminary metagenomics analysis

Metagenomics samples were processed through the pipeline shown in Supplemental Figure 1. Raw metagenomics genes were trimmed, filtered, and annotated, then assembled into gene and protein sequences using Metagenomics Analysis Toolkit (MOCAT2 v2.0.1).^40^ To determine the relative abundance of bacterial taxa for each sample, paired-end reads were analyzed using Metaphlan (v3.0.13) and results were graphed using ggplot2 (v3.3.5) in R (v4.1.2), as shown in Figure 2 and Supplemental Figures 2–5.^41,42^ MetaPhlan (v3.0.13) was also used to generate a Biological Observation Matrix (BIOM) for each sample, which was processed with QIIME2 (v2022.2.0) for diversity analyses.^41,43^ Alpha diversity was determined using the “diversity alpha” function and the Shannon Diversity Index parameter in QIIME2.^43^ Beta diversity was calculated using Bray–Curtis equilibrium distances and plotted with the Constrained Analysis of Principal Coordinates ordination method using the “capscale” function within the Vegan (v2.5–7) package in R (v4.1.3), as described previously.^44^ The Bray-Curtis distances were compared via PERMANOVA to assess differences in species-level composition between kidney transplant recipients and healthy individuals.^44^

### Metagenomics gene abundance

Nucleotide sequences were used to generate gene indices and Sequence Alignment Maps (SAM) using Bowtie2 (v2.4.1).^45^ Amino acid and nucleotide sequences were used to generate Generic Feature Format (GFF) files using PRODIGAL (v2.6.3).^46^ The paired genes from the SAMs were assigned to genes within the GFF files and counted using the SUBREAD package featureCounts (v2.0.0).^47^ Read counts were converted to relative counts based on the total count scaling factor to account for differences in read numbers between samples, using the formula below as previously described.^24,48^ Relative count was determined as follows:

$$\begin{array}{rl} & Relative\;Count\\ & \;\;\;\;\;\;\;=Log_{10}[(\frac{Gene\;Read\;Count}{Total\;Assigned\;Reads\;in\;Sample}\\ & \;\;\;\;\;\;\;\;\;\;\;\;\times \frac{Total\;Assigned\;Reads\;Across\;all\;Samples}{Number\;of\;Samples})+1] \end{array}$$

To assess gene length bias in read counts, relative counts were plotted as a function of gene length. To negate the bias, we further scaled the relative counts using the slope of the gene abundance linear regression as follows to reach the final normalized gene abundance:

$$\begin{array}{rl} & Normalized\;Gene\;Abundance\;=\;Relative\;Count\\ & \;\;\;\;\;\;\;\;\;\;\;\;\;\;\;\;\;\;\;\;\;\;\;+[Slope\;of\;Read\;Abundance\;Regression\\ & \;\;\;\;\;\;\;\;\;\;\;\;\;\;\;\;\;\;\;\;\;\;\;\times (Average\;Gene\;Length-Gene\;Length)] \end{array}$$

### Identification and characterization of GUS sequences

Metagenomic amino acid sequences were each aligned pairwise to 17 representative GUS enzymes with reported crystal structures using Protein–Protein BLAST (BLASTP v2.5.0+).^49^ Candidate sequences with >25% identity to any representative GUS enzyme were then assessed for the presence of seven conserved residues.^24^ Sequences that both met the identity threshold and contained all seven conserved residues were accepted as GUS enzymes. Accepted sequences were filtered for redundancies at a sequence identity threshold of 90% using CD-HIT (v4.8.1), and the output was used to form a representative set of GUS sequences for downstream analysis.^50^ Accepted sequences were aligned to representative sequences from each loop class in a Multiple Sequence Alignment (MSA), and GUS class was assigned according to parameters reported previously.^24–26^ Taxonomy was assigned to representative GUS sequences using BLASTP (v2.5.0+) as reported previously, and taxonomic identifiers were used to rename these sequences.^24,49^ Representative sequences were clustered using the EMBL-EBI search, which was combined with the GUS class and taxonomy to create cladograms using ggtree (v3.2.1) and ggplot2 (v3.3.5) in R (v4.1.2).^42,51,52^ Normalized gene abundances for GUS sequences were mapped to their corresponding GUS class then summed to form the plots in Figure 3c and Supplemental Figure 7. All relevant data including gene sequences, loop class, detailed taxonomy, and final gene counts can be found in Supplemental Table 2. Gene_IDs can be matched to their corresponding “Manuscript ID” using the “Gene_Barcode_Identifiers” tab.

### Complex protein lysate preparation

Human fecal samples were processed as previously described (21). Five to ten grams of thawed fecal material collected from each donor was resuspended in 25 mL cold extraction buffer (25 mM HEPES pH 6.5, 25 mM NaCl, one Roche Complete EDTA-free protease inhibitor tablet in 50 mL buffer) and 500 mg autoclaved garnet beads then vortexed. Samples were centrifuged at 300 x *g* for 5 min at 4°C and supernatant was collected. Then, 25 mL cold extraction buffer was added to the centrifuged pellet, which was again vortexed and centrifuged. Both supernatants were combined and centrifuged at 300 x *g* for 5 min at 4°C two additional times to further remove insoluble fiber. The supernatant was then sonicated twice on a Fischer Scientific Sonic Dismembrator Model 500 with 0.5 s pulses for 1.5 min and the lysate was mixed by inversion between each sonication. Lysate was then centrifuged at 17,000 x *g* for 20 min at 4°C to remove insoluble debris then decanted. The lysate was then concentrated with Amicon Ultra 15 mL 30 kDa centrifugal filters and exchanged with fresh extraction buffer three times to remove metabolites. After buffer exchanging, the total protein concentration of the final fecal lysate for each sample was measured with a Bradford assay using purified *Escherichia coli* β-glucuronidase as a reference standard. Complex protein lysates were aliquoted at 500 μL then flash frozen in liquid nitrogen and stored at −80°C until later use in proteomics and fecal lysate assays.

### GUS activity-based probe (ABP)

Cyclophellitol-based probe JJB397 was synthesized and purified as previously described to form a biotin-linked covalent inhibitor of GUS enzymes.^27^

### Metaproteomics

General Proteomics workflow is shown in Figure 4a and was adapted from our previously reported GUS-targeted Activity-Based Proteomic Profiling pipeline.^34^ Human fecal extracts (3 mg total protein) were thawed then incubated at 37°C for 60 min with 10 μM biotin-linked ABP JJB397 in 500 μL cold extraction buffer (25 mM HEPES pH 6.5, 25 mM NaCl, 1% DMSO final, one Roche Complete EDTA-free protease inhibitor tablet in 50 mL buffer). Reactions were quenched by adding 125 μL Triton + Urea buffer (50 mM HEPES pH 6.5, 125 mM NaCl, 1 mM EDTA, 1 mM EGTA, 1% v/v Triton X-100, 0.1% w/v SDS, DMSO final, 1:500 Roche Complete EDTA-free protease inhibitor tablet, 2 M urea) and heating at 95°C for 5 min. Samples were cooled on ice then washed five times with Triton + Urea buffer using 0.5 mL Amicon Ultra 10 K membrane filters to remove unreacted probe. Between each wash, samples were centrifuged at 13,000 x *g* for 5 min. The volume of each sample was then adjusted to 1 mL using Triton + Urea buffer. Twenty microliters per sample MyOne^TM^ Streptavidin T1 (Invitrogen) beads were washed three times in Triton + Urea buffer then diluted to a final volume allowing for 100 μL to be added to each sample. One hundred microliters of the Streptavidin T1 bead slurry was added to each 1 mL sample and the samples were then rotated end-over-end for 120 min at room temperature. Beads were then washed five times with 300 μL Triton + Urea buffer, five times with 300 μL 1 M NaCl, and five times with 100 μL NH_4_HCO_3_ (pH 7.8). Between each wash step, streptavidin beads were isolated using a DynaMag^TM^-2 Magnet (Invitrogen). After the final wash step, beads were resuspended in 100 μL NH_4_HCO_3_ (pH 7.8) and stored at −20°C for proteomic analysis. Samples were subjected to on-bead trypsin digestion, as previously described.^53^ After the last wash buffer step, 50 µl of 50 mM ammonium bicarbonate (pH 8) containing 1 µg trypsin (Promega) was added to beads overnight at 37°C with shaking. The next day, 500 ng of trypsin was added then incubated for an additional 3 h at 37°C with shaking. Supernatants from pelleted beads were transferred, then beads were washed twice with 50 μl LC/MS grade water. These rinses were combined with original supernatant, then acidified to 2% formic acid. Peptides were desalted with peptide desalting spin columns (Thermo) and dried via vacuum centrifugation. Peptide samples were stored at −80°C until further analysis.

### Metaproteomics LC-MS/MS analysis

The peptide samples analyzed were by LC-MS/MS using an Easy nLC 1200 coupled to a QExactive HF mass spectrometer (Thermo Scientific). Samples were injected onto an Easy Spray PepMap C18 column (75 μm id × 25 cm, 2 μm particle size) (Thermo Scientific) and separated over a 90 min method. The gradient for separation consisted of 5–40% mobile phase B at a 250 nl/min flow rate, where mobile phase A was 0.1% formic acid in water and mobile phase B consisted of 0.1% formic acid in 80% ACN. The QExactive HF was operated in data-dependent mode where the 15 most intense precursors were selected for subsequent fragmentation. Resolution for the precursor scan (*m*/*z* 350–1600) was set to 60,000 with a target value of 3 × 106 ions, 100 ms max injection time. MS/MS scans resolution was set to 15,000 with a target value of 5 × 104 ions, 60 ms max injection time. The normalized collision energy was set to 27% for HCD. Dynamic exclusion was set to 30 s, peptide match was set to preferred, and precursors with unknown charge or a charge state of 1 and ≥7 were excluded.

### Metaproteomics data analysis

Raw data were processed as described previously, with the following modifications.^21,23^ MaxQuant (v1.6.3.4) was used for peptide identification and quantitation.^54^ Data were searched against a reference database containing non-redundant protein sequences derived from metagenomic shotgun sequencing of all samples within the cohort (containing 645,141 entries), the Uniprot Human database (containing 26,122 entries), and a potential contaminants database.^55^ Proteins were filtered for a false discovery rate (FDR) of 1% at the unique peptide level, and potential contaminants and decoys were removed. Peptide peak areas were extracted and summed for each protein and the protein intensities were used for relative quantitation. Proteomic data can be found in Supplemental Table 3. Percent peptide coverage was compared for GUS enzymes identified using either the IGC or cohort metagenomics as the reference database using a Wilcoxon two-tailed t-test; results are shown in Supplemental Figure 11.^28^ Proteomic intensities were log_2_-transformed then compared between groups by Welch’s *t*-test; results are shown in Supplemental Figure 12. Normalized intensities were compared to normalized gene abundances as determined with metagenomics; *P* values reflect confidence in a slope that is significantly non-zero as determined by the Wald test and results are shown in Supplemental Figure 4. Normalized intensities were also correlated with normalized rate of MMF processing, with *P* values reflecting confidence in a slope that is significantly non-zero as determined by the Wald test; results are shown in Figure 4e,f, and Supplemental Figure 13.

### Fecal lysate MPAG activity

Thawed fecal lysate was diluted to 1 mg/mL in extraction buffer. Solid MPAG was purchased (Toronto Research Chemicals, CAT# M831520) and suspended in 100% DMSO at 50 mM and stored at −80°C until further use. MPAG (50 mM) was diluted in ddH_2_O to a working concentration of 4 mM MPAG. Final reaction conditions were 0.1 mg/mL fecal lysate and 400 µM MPAG in assay buffer (25 mM HEPES pH 6.5, 25 mM NaCl) at a final volume of 50 µL. And, 4 mM MPAG (5 µL) was added to 40 µL assay buffer and incubated at 37°C for 5 min. The reaction was initiated by adding 5 µL of fecal lysate (1 mg/mL) and incubated at 37°C. The reaction was quenched with an equivalent volume of 25% trichloroacetic acid at designated timepoints. Each sample had five endpoints (including 0 min) which were either every 15 or 30 min depending on the rate of reaction. Each quenched reaction was transferred to a 1.7-mL microcentrifuge tube and centrifuged at 13,000× g for 20 min. 80 µL of supernatant was transferred to a high-performance liquid chromatography (HPLC) vial and concentration of MPAG at each timepoint was quantified on an Agilent 1260 Infinity II liquid chromatography system. Samples were stored in an autosampler at 8°C prior to separation on an Agilent InfinityLab Poroshell 120 C18 column (4.6 × 150 mm, 2.7 µm) at 38°C with a flow rate of 0.9 mL/min and injection volume of 40 µL. The LC solvents were as follows; Solvent A: ddH_2_O with 0.1% formic acid; Solvent B: 98% acetonitrile with 0.1% formic acid. The LC flow gradient was as follows: constant 98% A/2% B for 0–2 min, linearly ramp to 2% A: 98% B over 2–15 min, constant 2% A: 98% B for 15–23 min, linearly ramp to 98% A: 2% B for 23–25 min. MPAG was monitored at 280 nm with a reference of 360 nm, eluting at 9.6 min. The area under the curve of MPAG was converted to concentration of MPAG using a standard curve. MPAG concentration as a function of reaction time was fit to a linear regression, with the slope representing reactivation of MPAG in nM/s. The slopes of three biological replicates were averaged to reach final rates in nM/s then log_2_ transformed. Rates were plotted in GraphPad PRISM GraphPad Prism (v9.3.1) with error bars representing standard error, shown in Figure 4b. Rates were then correlated to relative abundance of bacteria at all taxonomic levels, with *P* value reflecting confidence in a slope that is significantly non-zero as determined by the Wald test (Supplemental Table 4). Rates were correlated to relative abundance of bacteria at all taxonomic levels, with *P* values reflecting confidence in a slope that is significantly non-zero as determined by the Wald test; results are tabulated in Supplemental Table 4. Rates were also correlated with relative normalized abundance of GUS genes, with *P* values reflecting confidence in a slope that is significantly non-zero as determined by the Wald test; results are shown in Supplemental Figure 10. Rates were also correlated with normalized abundance of GUS proteins, with *P* values reflecting confidence in a slope that is significantly non-zero as determined by the Wald test; results are shown in Figure 4e,f and Supplemental Figure 13.

### Protein Modeling

The complete amino acid sequences for the five FMN-binding GUS enzymes detected using metaproteomics were modeled as monomers using Alphafold (v2.0).^29^ The models were aligned to *Roseburia hominis* 2 GUS (PDB: 6MVH) using the cealign plugin in PyMOL (v2.5.2).^26^ MPAG was manually docked into the active site in PyMOL using the crystal structure of *Eubacterium eligens* beta-glucuronidase bound to glucuronic acid (PDB: 6BJQ) overlaid to the structures of FMN-Binding GUS enzymes (PDB: 6MVH and PDB: 6MVG) as a reference scaffold.^26,56^

### Protein expression and purification

All GUS genes were codon-optimized for *E. coli* expression, synthesized, and ligated into a pLIC-His vector, then purchased from Bio Basic. The vectors were each transformed into chemically competent BL21-Gold (DE3) *E. coli* cells were grown on LB agar with ampicillin (100 μg/mL) at 37°C overnight. A single colony was selected and grown overnight in 100 mL of LB broth with ampicillin (100 μg/mL) at 37°C and vigorous shaking. After reaching saturation, 50 mL of the culture was added to 1 L of LB broth with ampicillin (100 μg/mL), ~3 μL Antifoam 204, and 500 μM FMN. The culture was incubated at 37°C and vigorously shaken until it reached an OD of 0.6 at 600 nm. After reaching the target OD, 1-thio-β-D-galactopyranoside (IPTG; 100 μM) was added to induce protein expression, the temperature was lowered to 18°C, and the culture was incubated overnight. The cells were collected by centrifugation at 4,500 × *g* at 4°C in a Sorvall (model RC-3B). Cell pellets were resuspended in 35 mL Purification Buffer A (20 mM Potassium Phosphate, 50 mM imidazole, 500 mM NaCl, and 50 μM FMN for FMN-binding enzymes, various pH) with DNase, lysozyme, and a complete-EDTA free protease inhibitor tablet (Roche). Resuspended cells were sonicated and clarified via centrifugation at 17,000 × *g* for 60 min in a Sorvall (model RC-5B). The lysate was flowed over a Ni-NTA HP column (GE Healthcare) then loaded onto the Aktaxpress FPLC system (Amersham Bioscience) and washed with Purification Buffer A. Protein was eluted with Buffer B (20 mM Potassium Phosphate, 250 mM Imidazole, 500 mM NaCl, 50 μM FMN, various pH). Fractions containing the protein of interest were concatenated then passed through a HiLoad 16/60 Superdex 200 gel-filtration column (GE Life Sciences). Protein was eluted in S200 buffer (20 mM HEPES, 50 mM NaCl, 50 μM FMN for FMN-binding enzymes, pH 8.0). Fractions containing the protein of interest were analyzed via SDS-PAGE, then those with >95% purity were combined and concentrated to ~10 mg/mL using 70 kDa cutoff molecular weight centrifuge concentrators (EMD Millipore). Samples were snap-frozen using liquid nitrogen and stored at −80°C.

### Site-directed mutagenesis

The mutant enzyme was created by placing a stop codon following residue 644, with primers for Rh2 Stop644 being synthesized by Integrated DNA Technologies. Primer sequences are listed in Supplemental Table 1. Rh2 Stop644 was produced as previously described, with the variant plasmid sequenced by EtonBio to confirm mutations.^20^

### In vitro MPA-G-specific activity

Solid MPA-G was purchased (Toronto Research Chemicals, CAT# M831520) and diluted in 100% DMSO to 50 mM and frozen at −80°C until later use. Working 4 mM solution of MPA-G was created by diluting the 50 mM DMSO stock in ddH_2_O. *In vitro*-specific activity (s^−1^) experiments were carried out in COSTAR 96-well, half area, clear, UV transparent assay plates. Reaction conditions consisted of MPAG (50 μM), enzyme (30–100 nM), and buffer (50 mM Na Acetate, 50 mM NaCl pH 4.0–6.0 or 50 mM HEPES, 50 mM NaCl pH 6.5–7.4) at a final volume of 50 µL. All reagents except enzyme were added to the mixture then preincubated at 37°C for 10 min. Enzyme was added to initiate the reaction, then the plate was incubated 37°C and absorbance was measured at 310 nm every 15 s over 50 min using a BMG LABTECH CLARIOstar plate reader. Production of MPA was measured after addition of GUS, which was fit by a custom linear regression analysis program in MATLAB to determine specific activity. Values shown in Figure 5e represent the averages of three biological replicates with error bars representing the standard error of mean (SEM). Tukey’s multiple comparisons test was used to assign statistical significance (*P < .05, ***P < .001, ****P < .0001, ns = non-significant).

## Supplementary Material

Supplemental Material

## Data Availability

Metagenomic and metaproteomic sequencing data derived from the kidney transplant patients will be made available at accession number phs001879 in the database of Genotypes and Phenotypes (dbGaP) upon publication.^57^ Approval from a local institutional review board approval will be needed to obtain the data. Sequencing data derived from healthy individuals will be made available at the Integrated Data Management and Comparative Analysis System for Microbial Genomes and Microbiomes Database (IMG/MER) upon publication.^58^ The metaproteomics data and protein sequence database for healthy individuals [will be] deposited to the ProteomeXchange Consortium via the PRIDE repository.^59^
