# Supplementary material for: Metagenomics combined with activity-based proteomics point to gut bacterial enzymes that reactivate mycophenolate
Source: Gut Microbes. 2022 Aug 11;14(1):2107289. doi: 10.1080/19490976.2022.2107289 (PMC9377255; doi:10.1080/19490976.2022.2107289)
Supplement: Supplemental Material [file KGMI_A_2107289_SM0613.zip › KGMI_A_2107289 supplementary .pptx]

## Slide 1
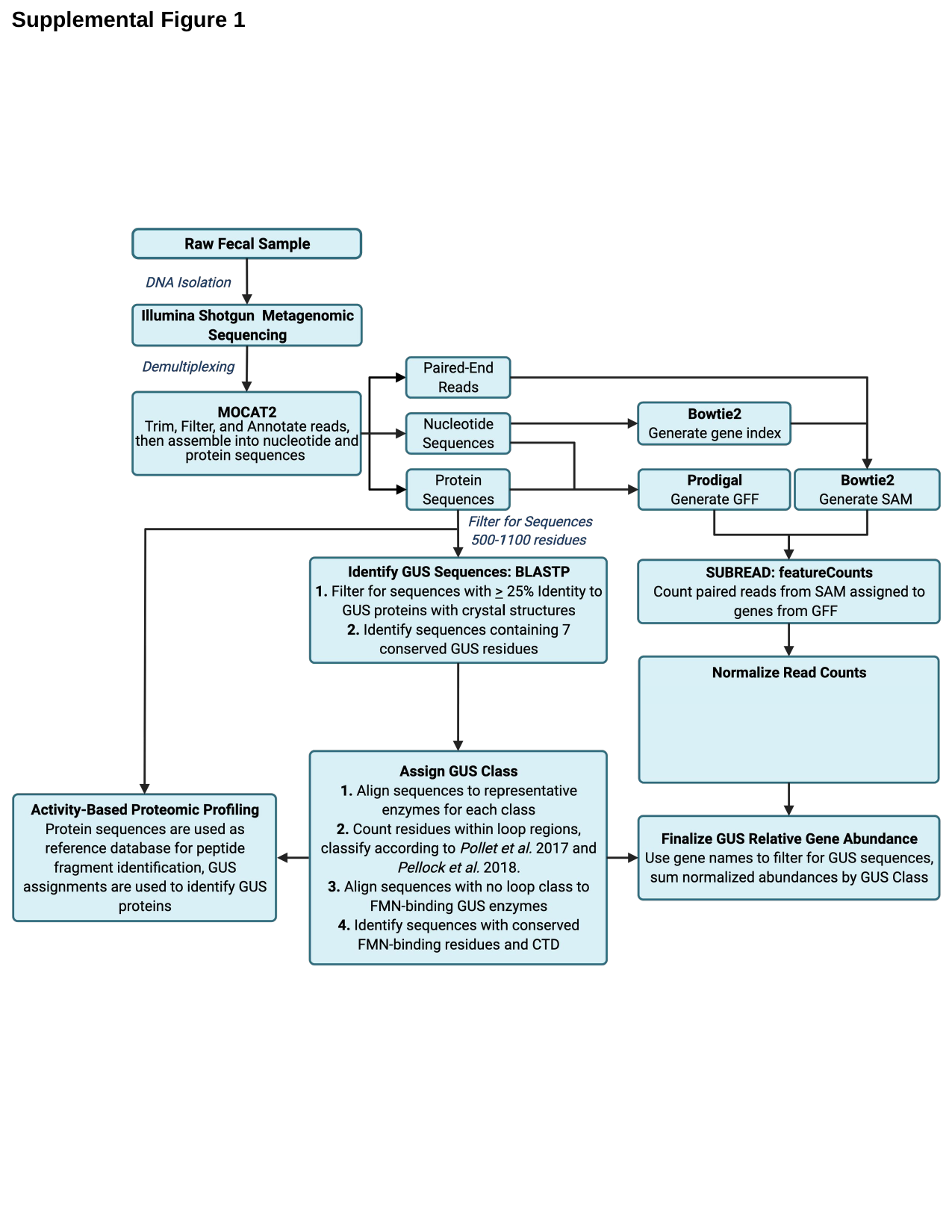

Supplemental Figure 1

## Slide 2
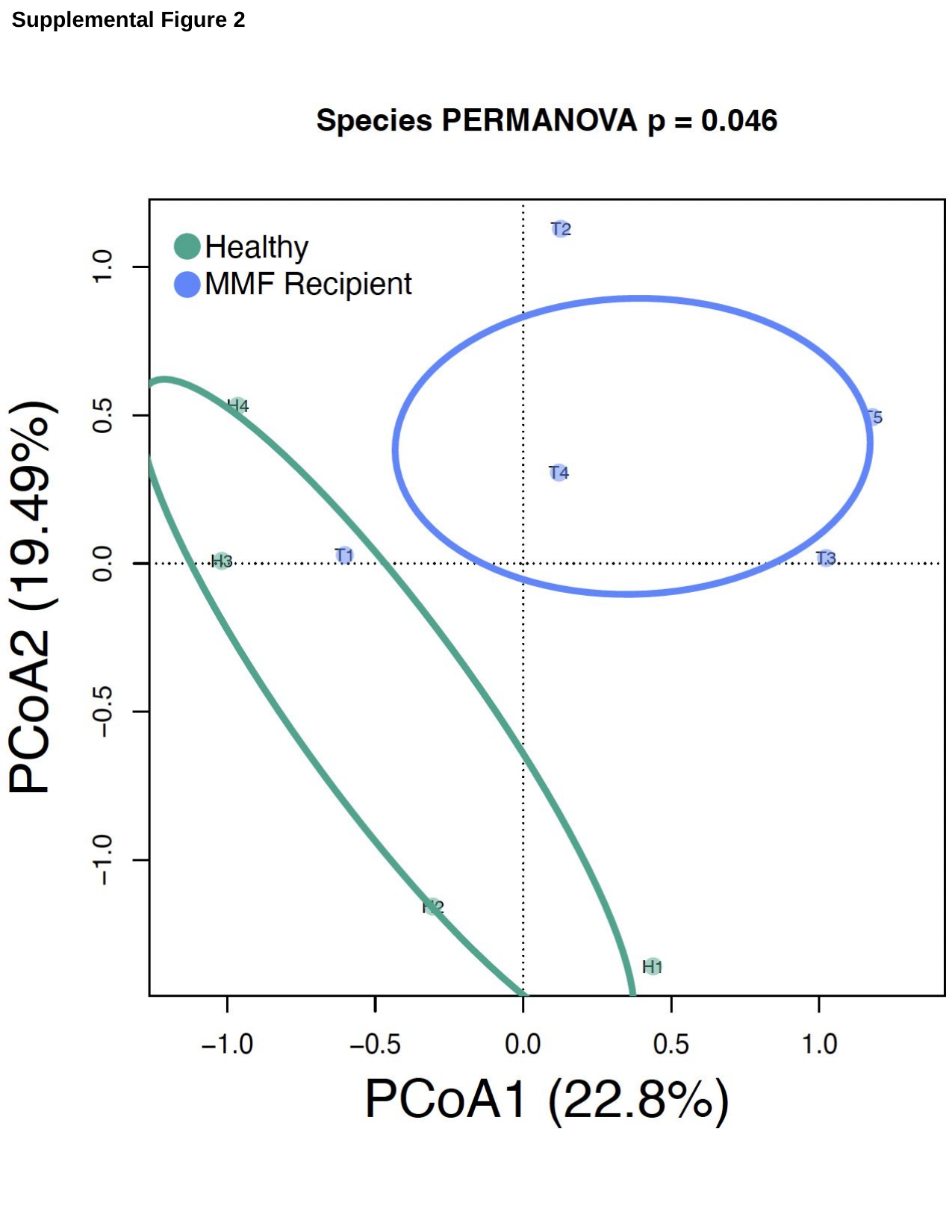

Supplemental Figure 2

## Slide 3
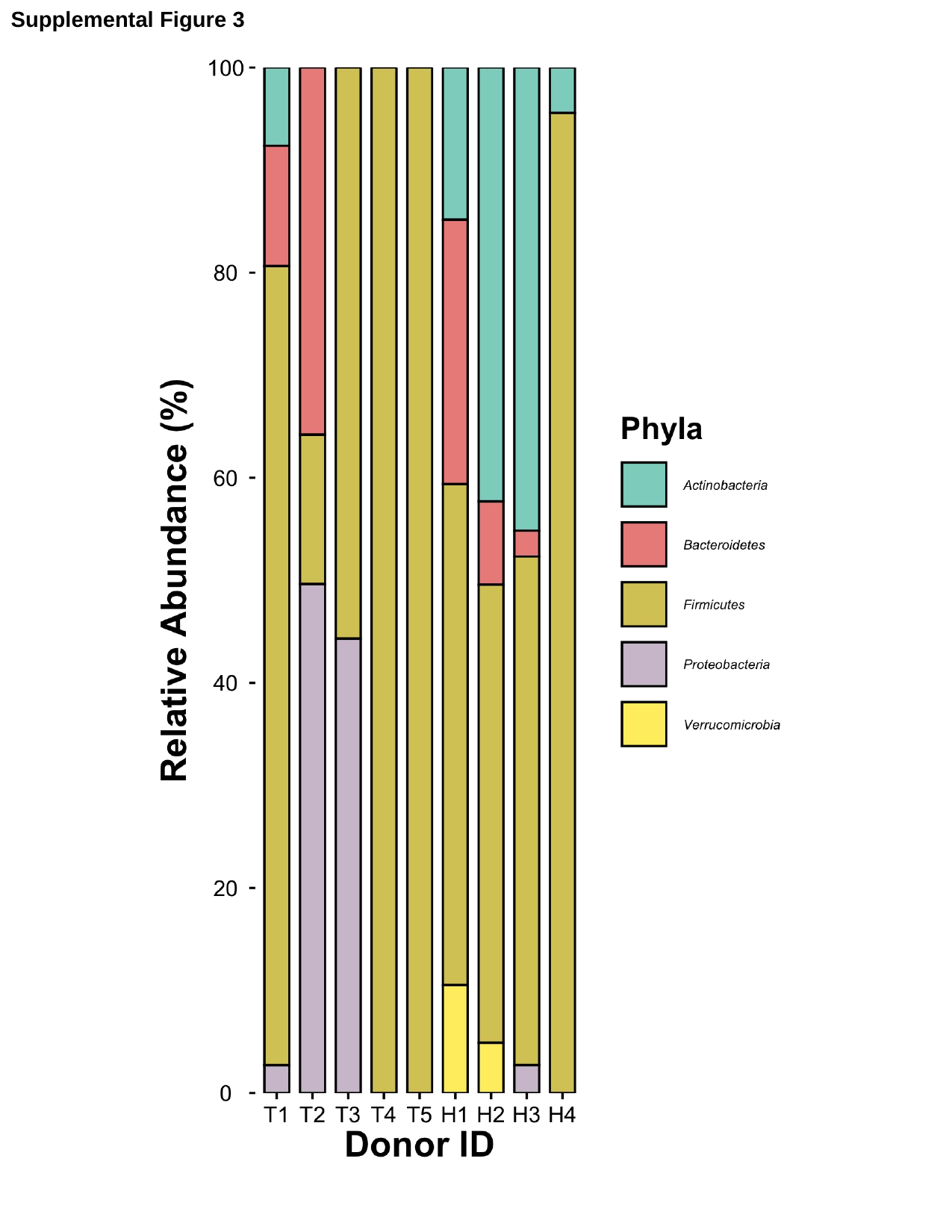

Supplemental Figure 3

## Slide 4
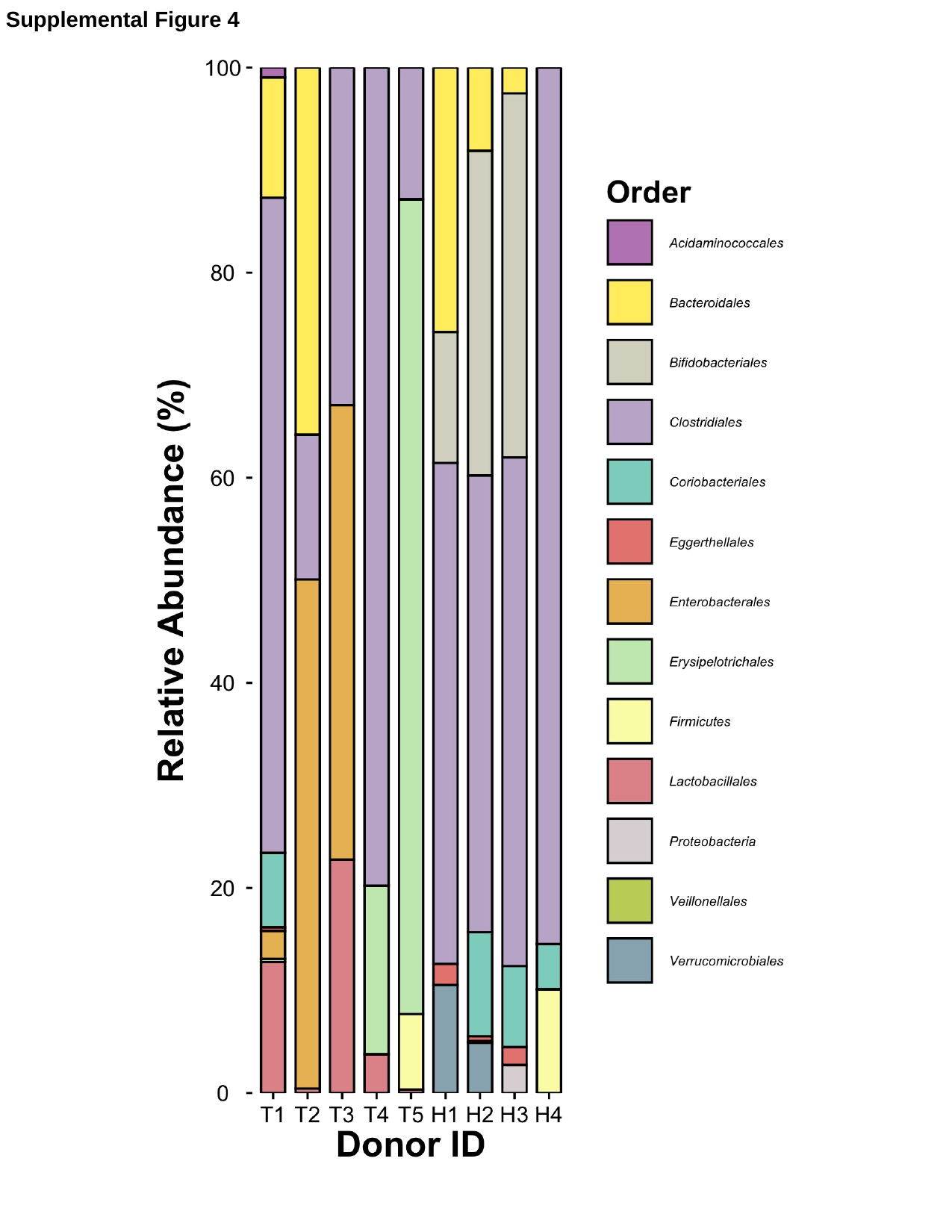

Supplemental Figure 4

## Slide 5
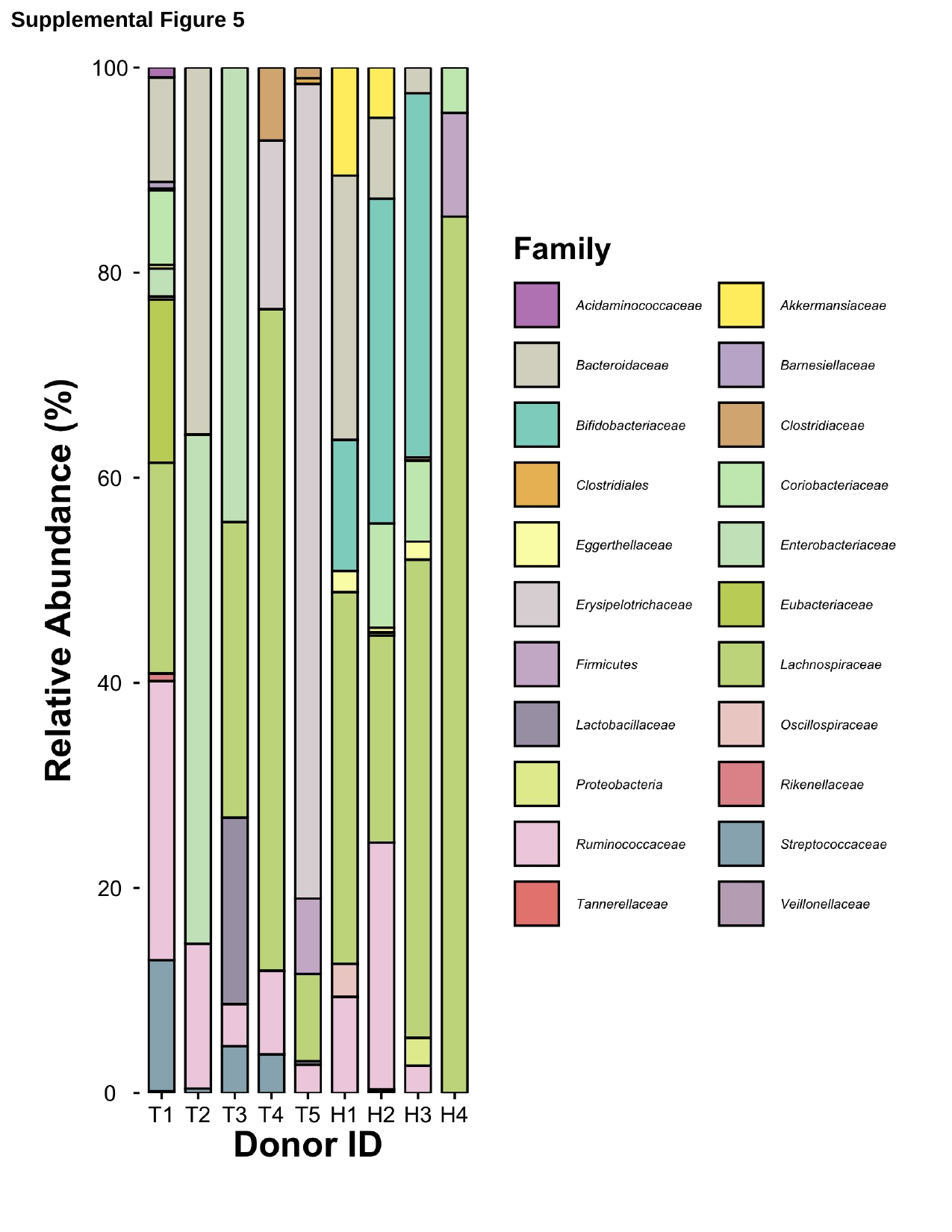

Supplemental Figure 5

## Slide 6
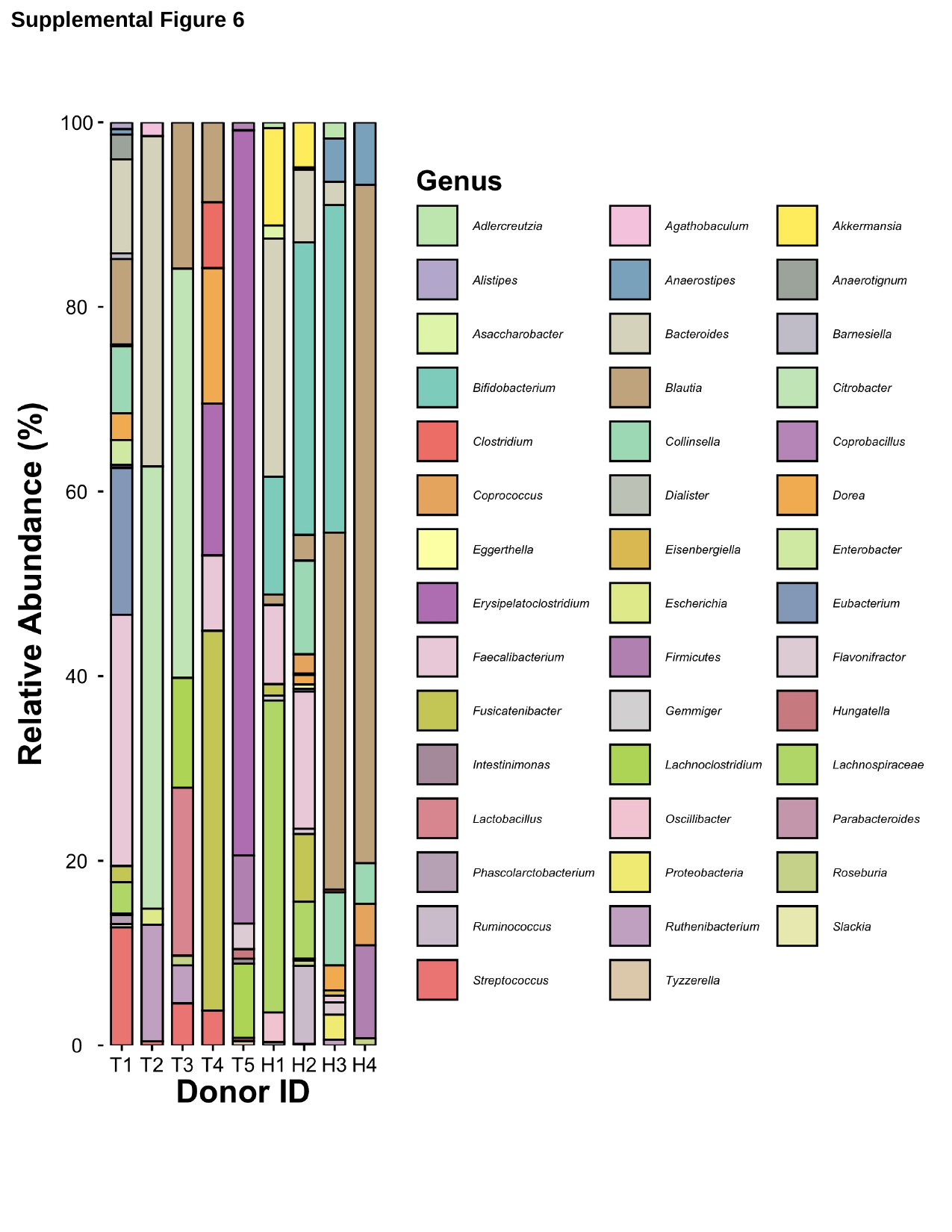

Supplemental Figure 6

## Slide 7
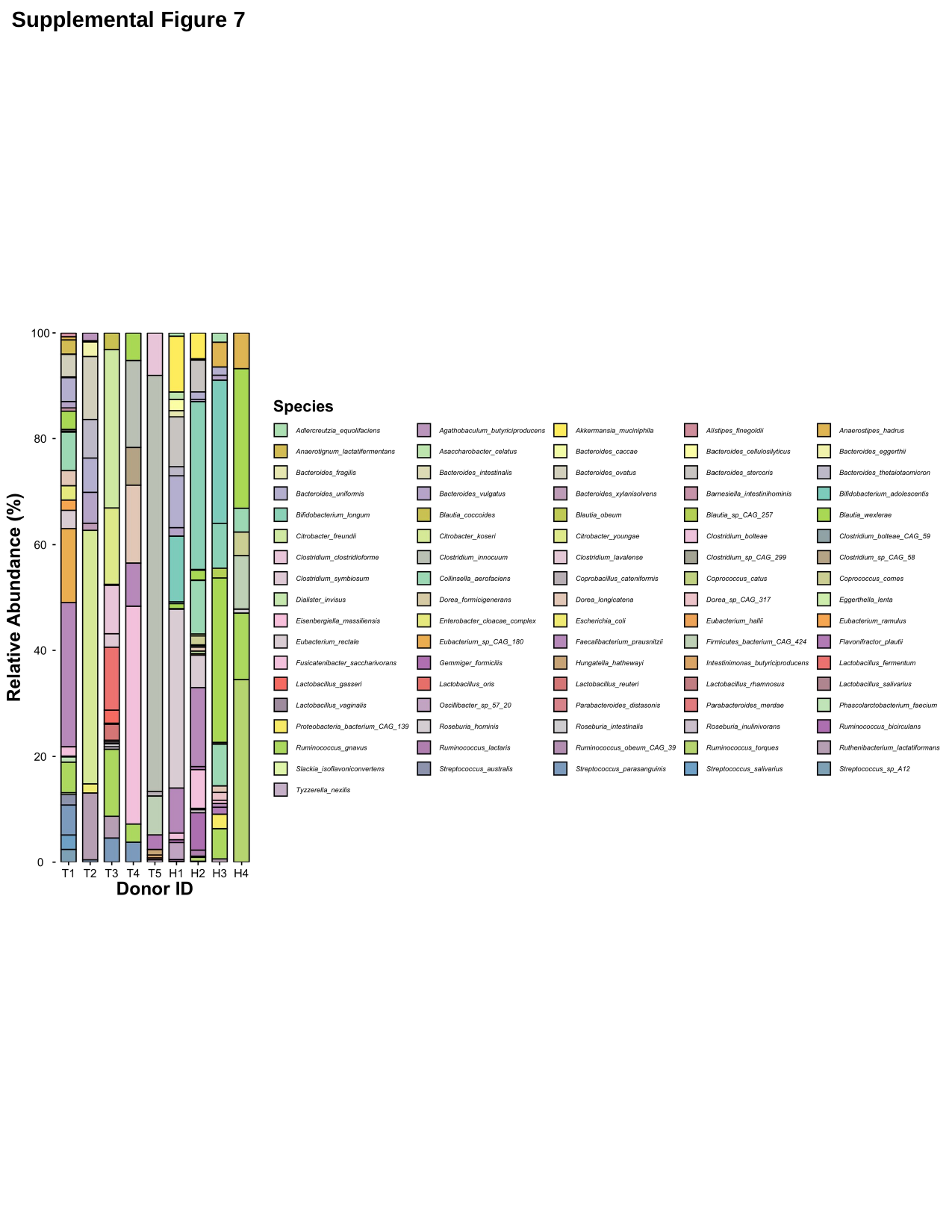

Supplemental Figure 7

## Slide 8
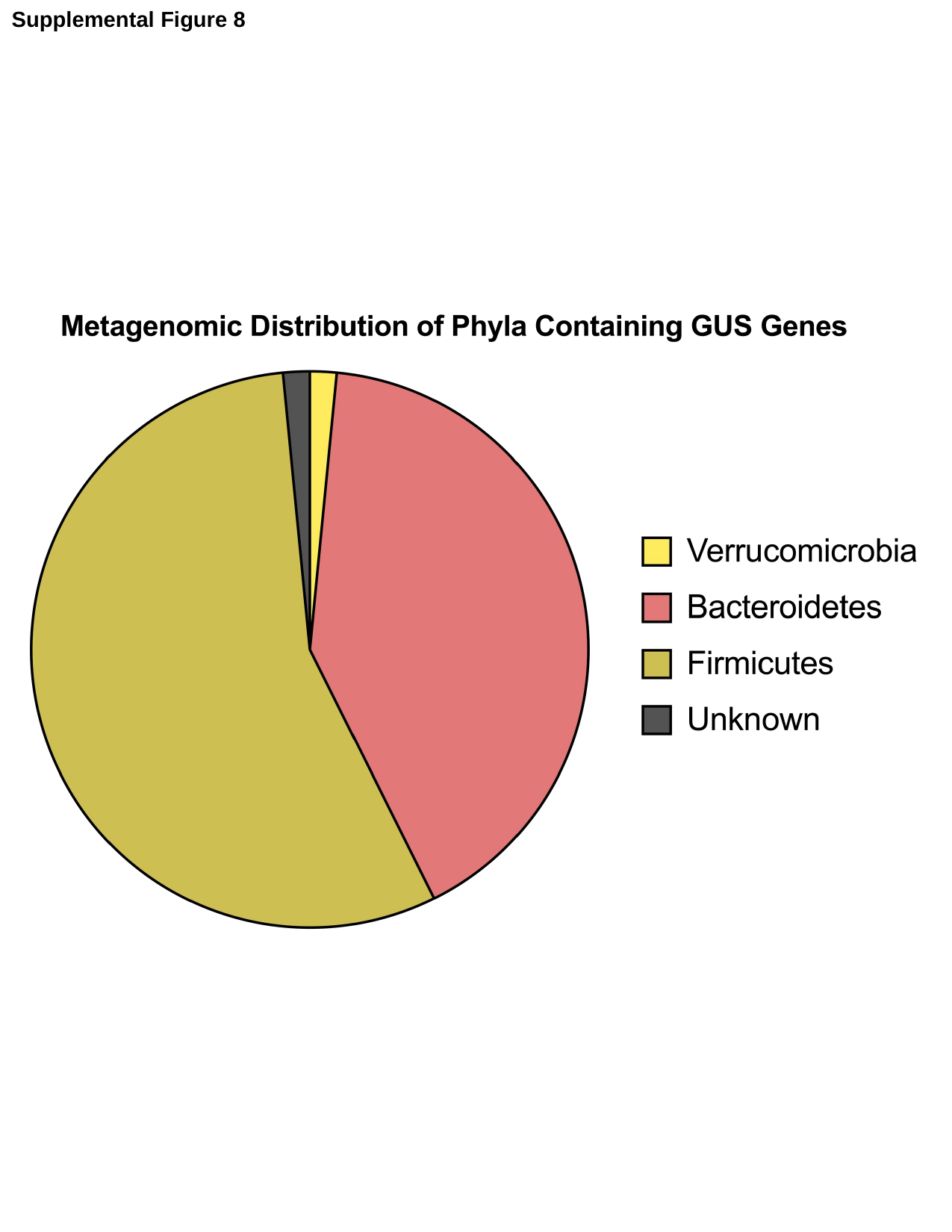

Supplemental Figure 8

## Slide 9
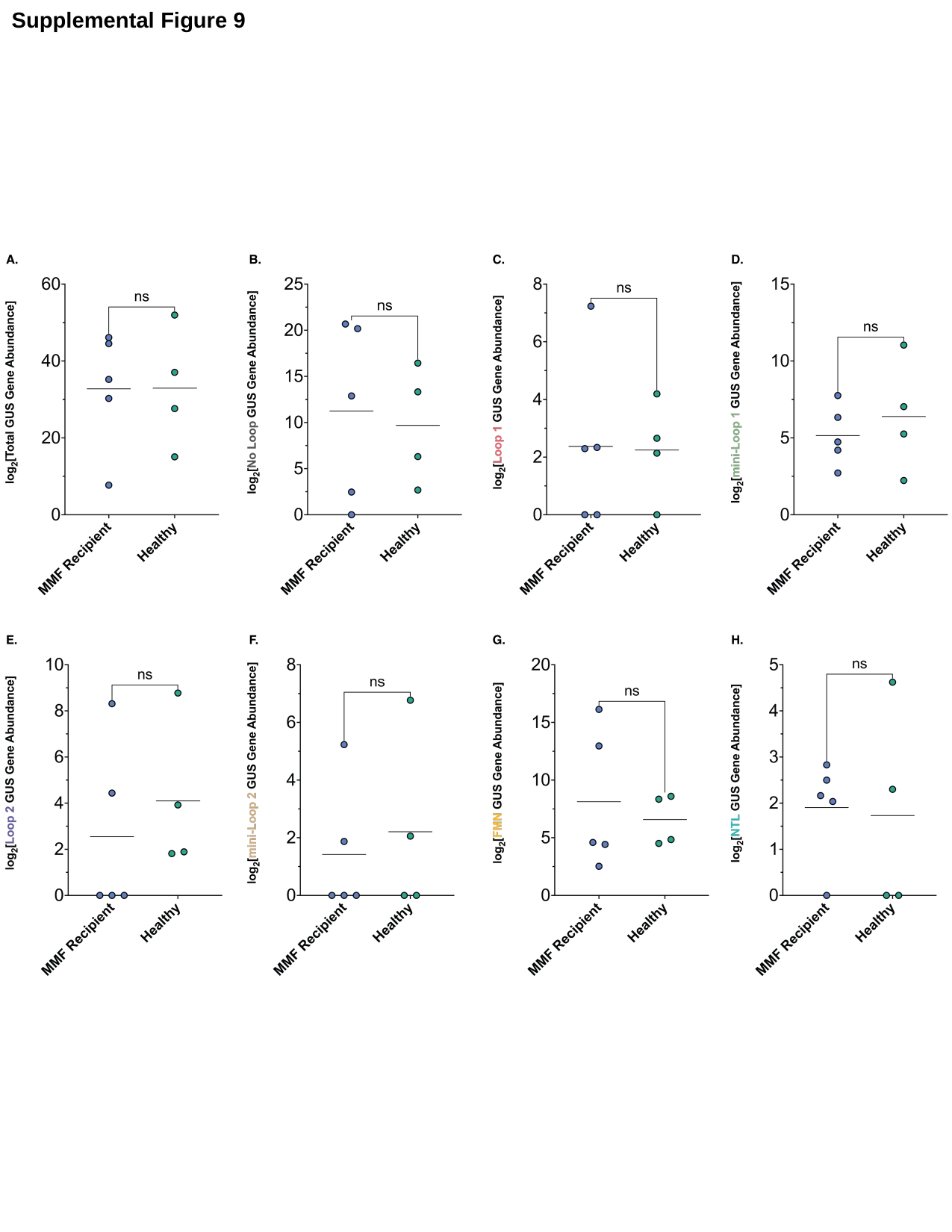

Supplemental Figure 9

## Slide 10
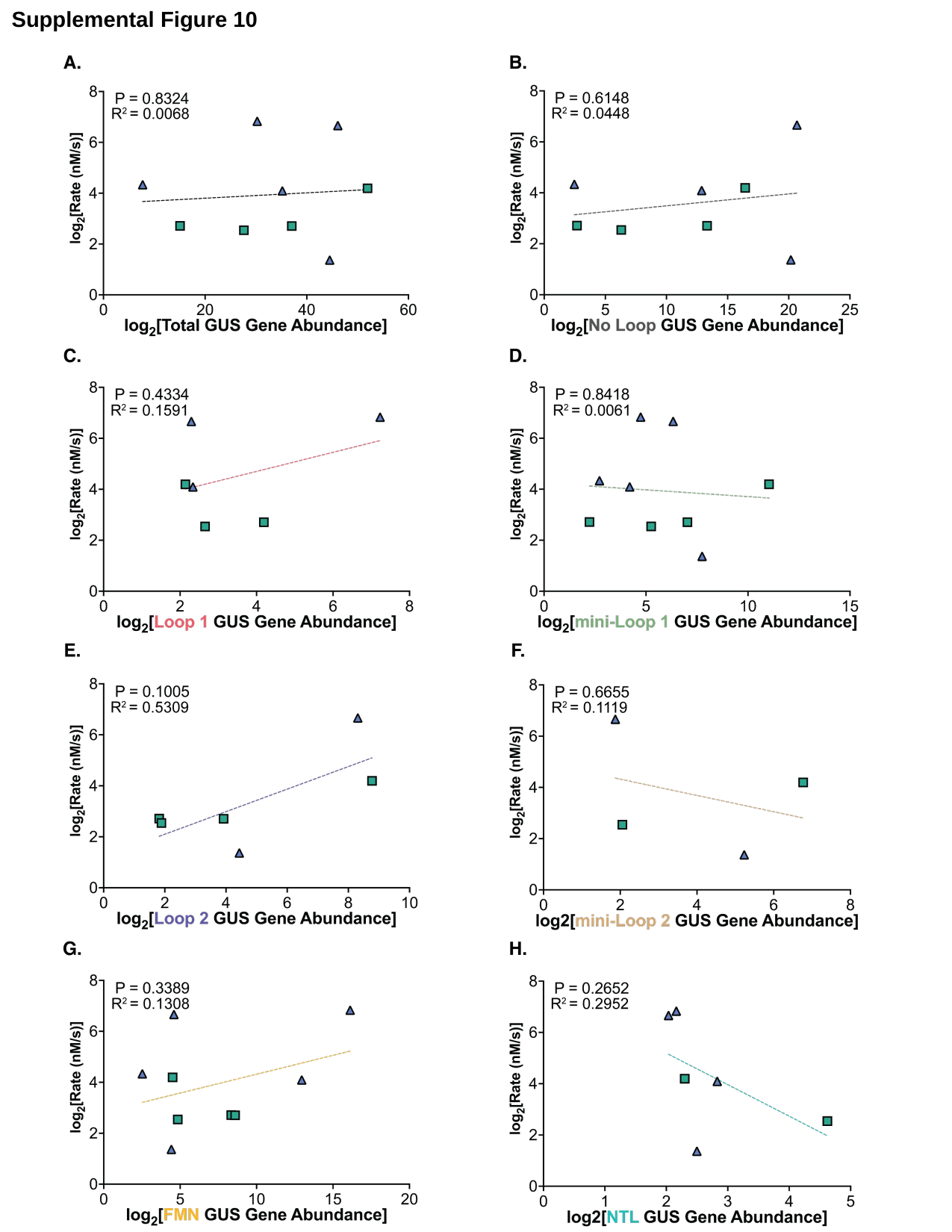

Supplemental Figure 10

## Slide 11
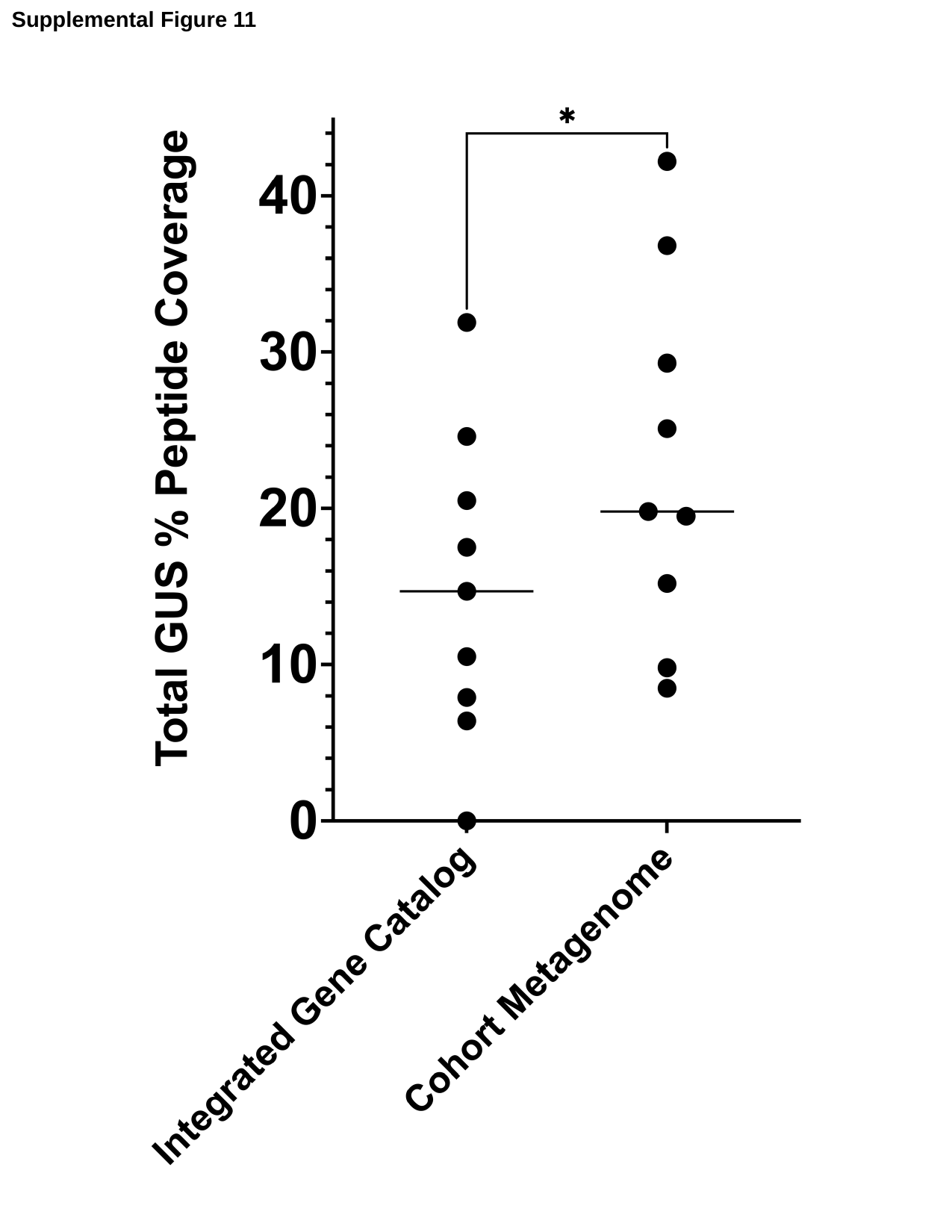

Supplemental Figure 11

## Slide 12
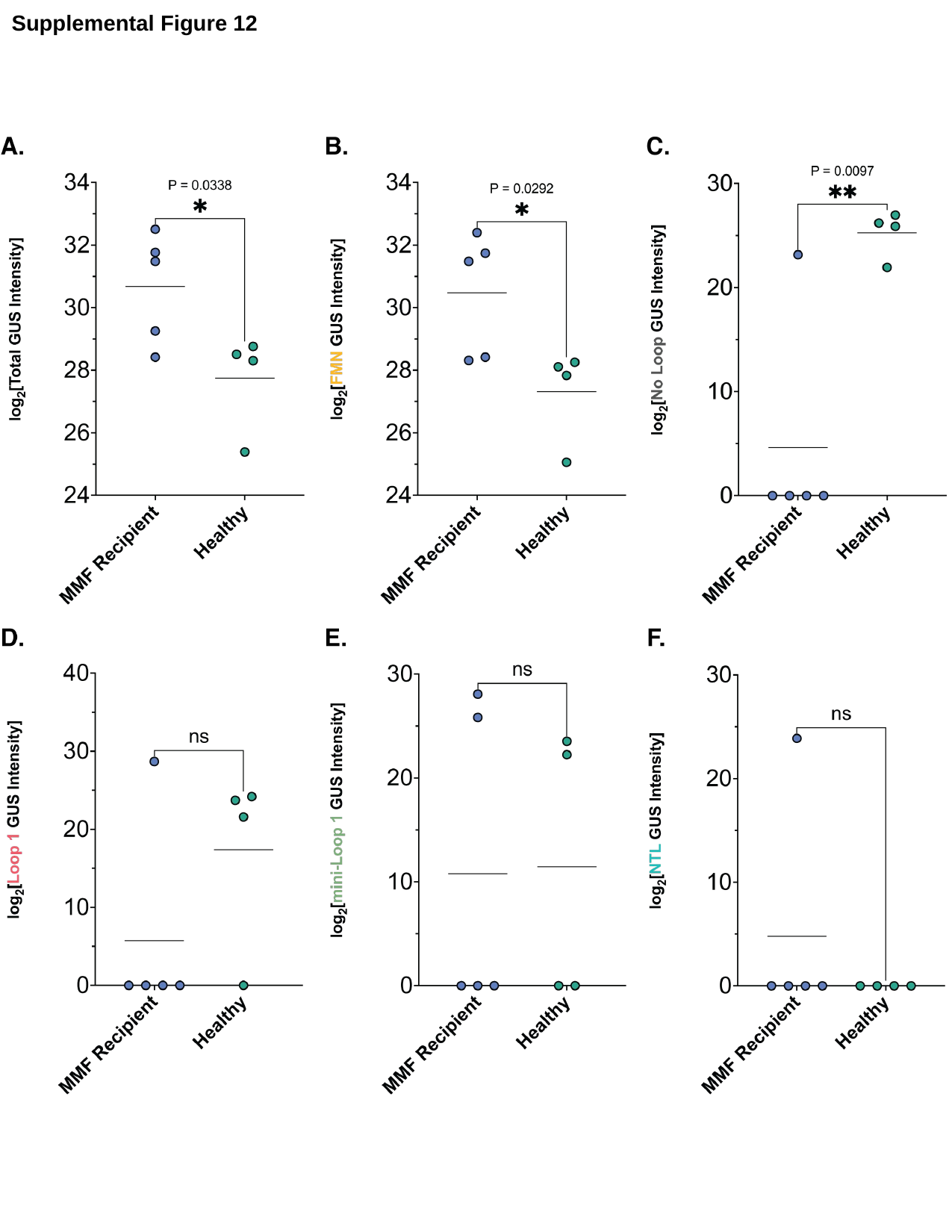

Supplemental Figure 12

## Slide 13
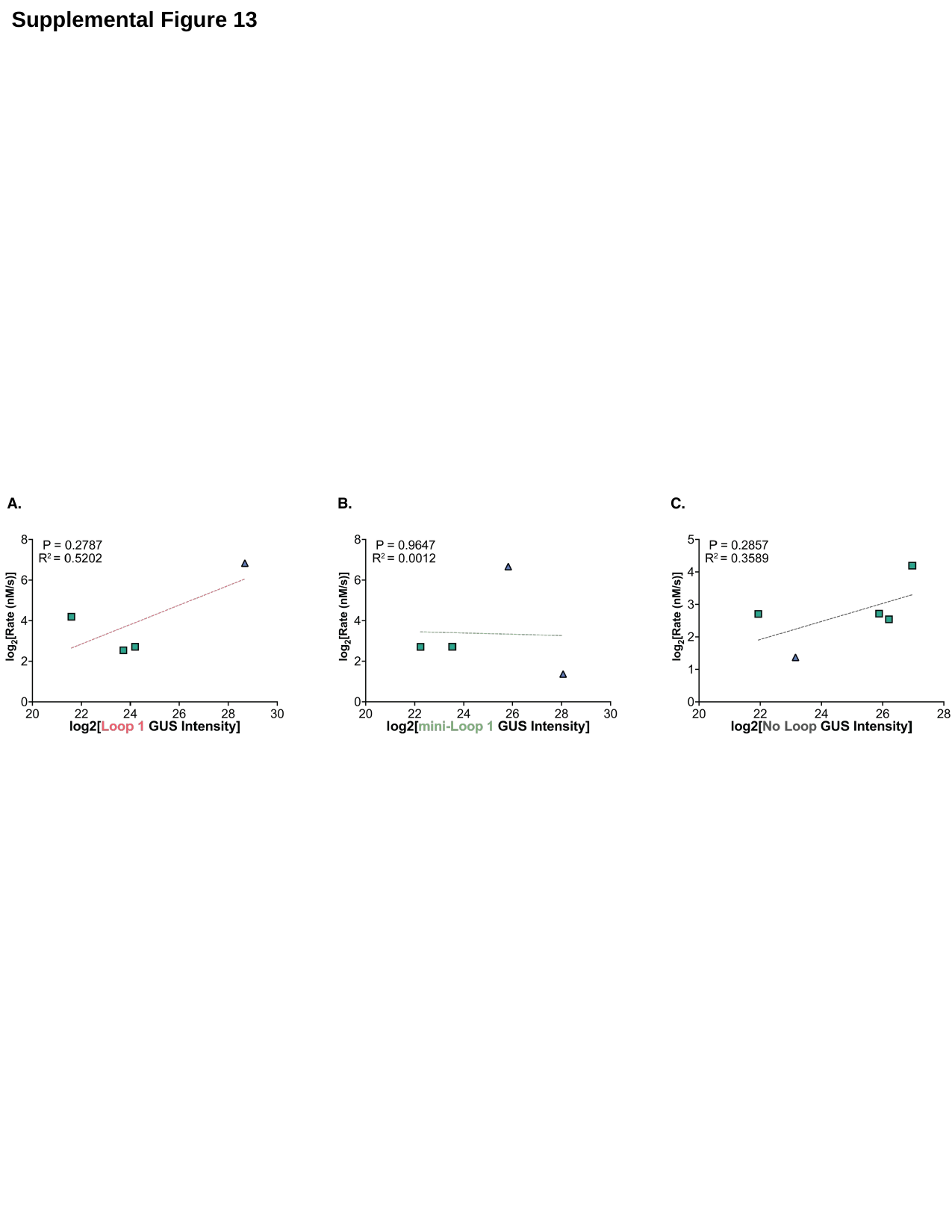

Supplemental Figure 13

## Slide 14
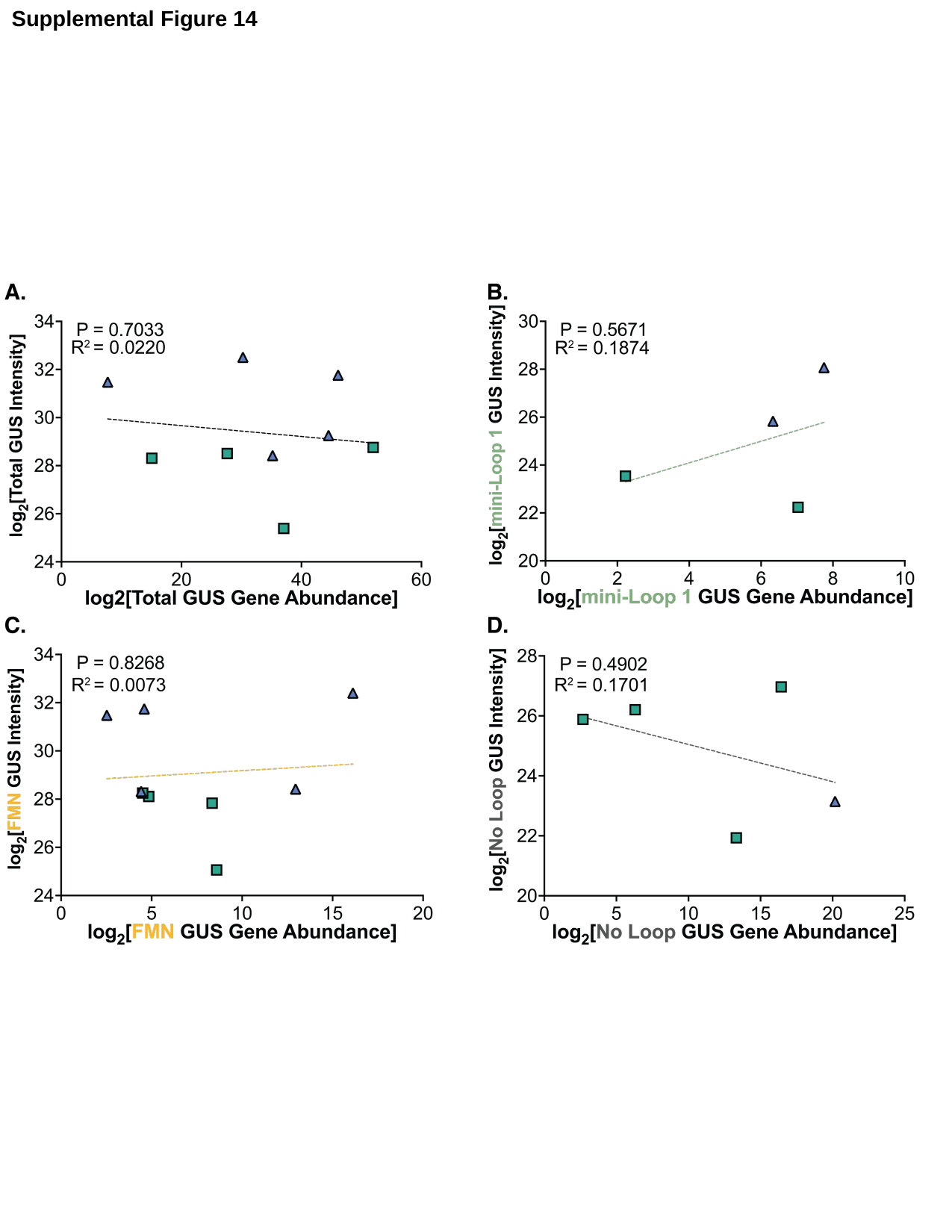

Supplemental Figure 14
